# Supplementary material for: Bicomponent nano- and microfiber aerogels for effective management of junctional hemorrhage
Source: Nat Commun. 2025 Mar 11;16:2403. doi: 10.1038/s41467-025-57836-0 (PMC11893793; doi:10.1038/s41467-025-57836-0)
Supplement: Supplementary file 1 — Supplementary Information [file 41467_2025_57836_MOESM1_ESM.pdf]

Supplementary Information

Bicomponent nano- and microfiber aerogels for effective management of junctional hemorrhage

S. M. Shatil Shahriar[1,2,#], Syed Muntazir Andrabi[1,#], Al-Murtadha Al-Gahmi[3,#], Zishuo Yan[1], Alec D. McCarthy[1], Chenlong Wang[1], Zakariya A. Yusuf[1], Navatha Shree Sharma[1], Milton E. Busquets[1,4], Mallory I. Nilles[1], Carlos Poblete Jara[5], Kai Yang[6], Mark A. Carlson[3,*], & Jingwei Xie[1,7*]

[1]Department of Surgery - Transplant and Mary & Dick Holland Regenerative Medicine Program, College of Medicine, University of Nebraska Medical Center, Omaha, NE, USA

[2]Eppley Institute for Research in Cancer and Allied Diseases, College of Medicine, University of Nebraska Medical Center, Omaha, NE, USA

[3]Department of Surgery - General Surgery, College of Medicine, University of Nebraska Medical Center, Omaha, NE, USA

[4]Pancreatic Cancer Center of Excellence, University of Nebraska Medical Center, Omaha, NE, USA

[5]Department of Surgery - Vascular Surgery, College of Medicine, University of Nebraska Medical Center, Omaha, NE, USA

[6]Department of Surgery - Plastic & Reconstructive Surgery, College of Medicine, University of Nebraska Medical Center, Omaha, NE, USA

[7]Department of Mechanical and Materials Engineering, University of Nebraska Lincoln, Lincoln, NE, USA

[*]e-mail: macarlso@unmc.edu (M. A. Carlson); jingwei.xie@unmc.edu (J. Xie)

[#]Contribute equally.

Table of Contents

1. Supplementary Tables …………...………………………………………….. pages S3-S27
2. Supplementary Figures …...……...………………………………………... pages S28-S54
3. References ………………………………………………………………. pages S55-S56

Supplementary Tables

Supplementary Table 1. The ratio between the fixed volume (V_f) and the recovery volume (V_r) of samples. Data were presented as mean ± s.d., $n = 3$. The significant difference was detected by an unpaired t-test (XStat® vs aerogels). Source data are provided as a Source Data file.

**Hemostats**	**V_f (mm^3)**	**Vr (mm^3)**	**$V_f : V_r$**	***p* Value**
XStat®	419.21 ± 45.05	2513.28 ± 157.08	6.01 ± 0.37	Reference
NA	154.99 ± 44.13	2853.61 ± 119.97	19.60 ± 6.40	0.0214
MA	281.70 ± 99.37	3089.23 ± 45.35	11.87 ± 3.92	0.0615
NMA	198.18 ± 5.00	3115.41 ± 45.35	15.72 ± 0.56	0.0001

NA: nanofiber aerogels; MA: microfiber aerogels; NMA: nano- and microfiber aerogels.

Supplementary Table 2. Comparison of shape recovery rate of NMA in blood with all other hemostats reported in the literature known for shape-recovery properties in blood (**Fig. 4k**).

**No.**	**Hemostats Under Development**	**Shape Recovery Rate in Blood (s/cm)**		**References**
		Mean	s.d.	
**1**	NMA	0.90	0.14	This Work
**2**	MA	1.09	0.14	
**3**	NA	1.21	0.31	
**4**	XStat®	7.27	0.90	
**5**	Nanofiber Peanut (NFP)	2.63	0.32	1
**6**	MACS1	4.00	0.81	2
**7**	MACS2	2.50	0.40	
**8**	QCS/PDA0.5	72.40	11.10	3
**9**	QCS/PDA1	47.60	6.90	
**10**	QCS/PDA2	41.60	7.00	
**11**	QCS/PDA3	31.00	4.90	
**12**	QCS/PDA4	19.80	4.90	
**13**	GT25/DA0	26.20	1.10	4
**14**	GT25/DA2	23.55	1.60	
**15**	GT25/DA4	21.20	1.15	
**16**	GT25/DA6	12.10	0.85	
**17**	GT25/DA8	11.70	1.25	
**18**	GT25/DA10	12.00	0.80	
**19**	GCS	10.00	-	5
**20**	GCSF-1	10.00	-	
**21**	GCSF-2	6.000	-	
**22**	GCSF-3	5.000	-	
**23**	GCSF/CT	5.000	-	
**24**	CS20/PDA1.5	8.667	1.13	
**25**	CS20/PDA3	7.533	1.00	

**26**	CS20/PDA4.5	6.20	0.80	[6]
**27**	CS20/PDA6	5.13	0.40	
**28**	CS20/PDA7.5	4.47	0.80	
**29**	QCSG/CNT0	7.70	-	[7]
**30**	QCSG/CNT4	16.15	-	
**31**	CA1, 160 ºC, 12h	41.10	2.70	
**32**	CA2, 180 ºC, 10h	27.04	1.40	[8]
**33**	GS	306.50	18.32	
**34**	A-spCS	4.37	1.36	[9]
**35**	spCS	4.00	1.13	

NA: nanofiber aerogels; MA: microfiber aerogels; NMA: nano- and microfiber aerogel; MACS: microchannelled alkylated chitosan sponge; QCS/PDA: quarternized chitosan/polydopamine; GT/DA: gelatin/dopamine; GCS: glutaraldehyde/chitosan; GCSF: glutaraldehyde/chitosan/silk fibronin short nanofibers; GCSF/CT: glutaraldehyde/chitosan/silk fibronin short nanofibers with built in cargo-loaded $CaCO_3$ and protonated tranexamic acid; CS/PDA: chitosan/polydopamine; QCSG/CNT: glycidyl methacrylate-functionalized quarternized chitosan/carbon nanotube; CA: carbonized gelatin aerogel; GS: gelatin sponge; A-spCS: alkylated superporous chitosan sponge; spCS: superporous chitosan sponge.

Supplementary Table 3. *p* value table for comparison of the percentages of platelets and RBCs adhesion between samples (**Fig. 5c, d**). The significant difference was detected by two-way ANOVA with Tukey's multiple comparisons test. *n* = 3 samples.

Tukey's multiple comparisons test	Mean Diff.	95.00% CI of diff.	Below threshold?	Summary	P Value
Row 1:XStat® vs. Row 1:NA	-16.92	-20.80 to -13.05	Yes	***	0.0009
Row 1:XStat® vs. Row 1:MA	-10.01	-12.31 to -7.718	Yes	***	0.0009
Row 1:XStat® vs. Row 1:NMA	-37.95	-46.64 to -29.25	Yes	***	0.0009
Row 1:XStat® vs. Row 2:XStat®	-3.436	-11.91 to 5.043	No	ns	0.2590
Row 1:XStat® vs. Row 2:NA	-12.73	-24.48 to -0.9742	Yes	*	0.0428
Row 1:XStat® vs. Row 2:MA	-5.972	-15.32 to 3.380	No	ns	0.1169
Row 1:XStat® vs. Row 2:NMA	-42.82	-65.77 to -19.87	Yes	*	0.0148
Row 1:NA vs. Row 1:MA	6.912	5.328 to 8.496	Yes	***	0.0009
Row 1:NA vs. Row 1:NMA	-21.02	-25.84 to -16.21	Yes	***	0.0009
Row 1:NA vs. Row 2:XStat®	13.49	6.280 to 20.70	Yes	*	0.0148
Row 1:NA vs. Row 2:NA	4.196	-5.522 to 13.91	No	ns	0.2332
Row 1:NA vs. Row 2:MA	10.95	3.139 to 18.76	Yes	*	0.0257
Row 1:NA vs. Row 2:NMA	-25.90	-46.04 to -5.751	Yes	*	0.0306

Row 1:MA vs. Row 1:NMA	-27.93	-34.34 to -21.53	Yes	***	0.0009
Row 1:MA vs. Row 2:XStat®	6.576	-0.9382 to 14.09	No	ns	0.0646
Row 1:MA vs. Row 2:NA	-2.716	-13.14 to 7.708	No	ns	0.4938
Row 1:MA vs. Row 2:MA	4.040	-4.219 to 12.30	No	ns	0.1885
Row 1:MA vs. Row 2:NMA	-32.81	-54.06 to -11.56	Yes	*	0.0211
Row 1:NMA vs. Row 2:XStat®	34.51	26.20 to 42.82	Yes	**	0.0013
Row 1:NMA vs. Row 2:NA	25.22	16.16 to 34.28	Yes	**	0.0078
Row 1:NMA vs. Row 2:MA	31.97	23.60 to 40.35	Yes	**	0.0023
Row 1:NMA vs. Row 2:NMA	-4.875	-22.13 to 12.38	No	ns	0.4447
Row 2:XStat® vs. Row 2:NA	-9.292	-12.85 to -5.734	Yes	**	0.0089
Row 2:XStat® vs. Row 2:MA	-2.537	-3.508 to -1.565	Yes	**	0.0089
Row 2:XStat® vs. Row 2:NMA	-39.39	-54.47 to -24.30	Yes	**	0.0089
Row 2:NA vs. Row 2:MA	6.756	4.169 to 9.343	Yes	**	0.0089
Row 2:NA vs. Row 2:NMA	-30.09	-41.62 to -18.57	Yes	**	0.0089
Row 2:MA vs. Row 2:NMA	-36.85	-50.96 to -22.74	Yes	**	0.0089

NA: nanofiber aerogels; MA: microfiber aerogels; NMA: nano- and microfiber aerogels. Row 1: percentages of platelets adhesion; Row 2: percentages of RBCs adhesion; ns = a non-significant value of > 0.05.

Supplementary Table 4. Summary of physical characteristics**.**

Parameters	Weighting Factor	NA	MA	NMA
Open Pores (%)	0.14	60.45	93.91	91.75
Specific Elastic Modulus (MPa cm^3/ g)	0.18	9.81	10.34	28.54
Shape Memory (%)	0.23	49.70	70.50	100.00
Blood Absorption Rate (g/cm^3/s)	0.20	0.16	0.18	0.23
Adhered RBCs (%)	0.10	46.22	49.46	76.31
Adhered Platelets (%)	0.15	50.41	43.50	71.44
Total		216.70	267.90	368.20
Weighted Average		33.87	42.73	59.36

NA: nanofiber aerogels; MA: microfiber aerogels; NMA: nano- and microfiber aerogels.

Supplementary Table 5. Summary of *in vitro* hemostatic properties. The lower the values, the higher the degree of procoagulant activities.

**Weighting Parameters**	**Weighting Factor**	**NA**	**MA**	**NMA**
Shape Recovery Time in Human Blood (s)	0.30	6.70	6.00	5.00
BCI (%)	0.15	9.30	8.43	7.77
Blood Clotting Time (s)	0.15	36.67	40.00	25.00
PT (s)	0.15	21.66	25.00	11.70
aPTT (s)	0.15	20.00	33.33	15.00
Hemolysis (%)	0.10	0.42	0.38	0.38
Total		94.76	113.10	64.84
Weighted Average		15.19	17.85	10.45

BCI: blood clotting index; PT: prothrombin time; aPTT: activated partial thromboplastin time; NA: nanofiber aerogels; MA: microfiber aerogels; NMA: nano- and microfiber aerogels.

Supplementary Table 6. Weighted T score table of physical characteristics of aerogels.

**Parameters**	**Weighting Factor**	**NA**	**MA**	**NMA**
Open Pores	0.14	35.88	57.76	56.35
Specific Elastic Modulus	0.18	42.62	43.23	64.13
Shape Memory	0.23	38.51	48.59	62.88
Blood Absorption Rate	0.20	40.34	45.87	63.77
Adhered RBCs	0.10	41.76	44.16	64.07
Adhered Platelets	0.15	46.04	40.22	63.73
Total		245.10	279.80	374.90
Weighted Average		40.70	46.67	62.62

NA: nanofiber aerogels; MA: microfiber aerogels; NMA: nano- and microfiber aerogels.

Supplementary Table 7. Weighted T score table of *in vitro* hemostatic properties. The lower the values, the higher the degree of procoagulant activities.

**Weighting Parameters**	**Weighting Factor**	**NA**	**MA**	**NMA**
Shape Recovery Time in Human Blood	0.30	61.46	51.43	37.09
Blood Clotting Index	0.15	62.74	48.93	38.31
Blood Clotting Time	0.15	54.32	59.50	36.17
PT	0.15	53.91	59.81	36.27
aPTT	0.15	46.41	63.64	39.94
Hemolysis	0.10	64.04	44.38	41.57
Total		342.90	327.70	229.30
Weighted Average		57.45	54.65	37.89

PT: prothrombin time; aPTT: activated partial thromboplastin time; NA: nanofiber aerogels; MA: microfiber aerogels; NMA: nano- and microfiber aerogels.

Supplementary Table 8. The ARRIVE guidelines 2.0: author checklists.

**Item**		**Recommendation**	**Section/line number, or reason for not reporting**
Study design	1	For each experiment, provide brief details of study design including:<br>a. The groups being compared, including control groups. If no control group has been used, the rationale should be stated.<br>b. The experimental unit (e.g. a single animal, litter, or cage of animals).	a. Methods > animal studies > 4th paragraph.<br>b. A single animal
Sample size	2	a. Specify the exact number of experimental units allocated to each group, and the total number in each experiment. Also indicate the total number of animals used.<br>b. Explain how the sample size was decided. Provide details of any *a priori* sample size calculation, if done.	a. 5 swine/group and 20 swine/exp.<br>b. Methods > power analysis.
Inclusion and exclusion criteria	3	a. Describe any criteria used for including and excluding animals (or experimental units) during the experiment, and data points during the analysis. Specify if these criteria were established *a priori.* If no criteria were set, state this explicitly.<br>b. For each experimental group, report any animals, experimental units or data points not included in the analysis and explain why. If there were no exclusions, state so.<br>c. For each analysis, report the exact value of *n* in each experimental group.	a. No criteria were used for including or excluding animal.<br>b. No exclusion<br>c. n = 5 swine/group
Randomization	4	a. State whether randomization was used to allocate experimental units to control and treatment groups. If done, provide the method used to generate the randomization sequence.<br>b. Describe the strategy used to minimize	a. not done.<br>b. All animal were treated the same way, and the time of physical

		potential confounders such as the order of treatments and measurements, or animal/cage location. If confounders were not controlled, state this explicitly.	activity was always the same.
Blinding	5	Describe who was aware of the group allocation at the different stages of the experiment (during the allocation, the conduct of the experiment, the outcome assessment, and the data analysis).	Technicians and pathologists were not familiar with the study design.
Outcome measures	6	a. Clearly define all outcome measures assessed (e.g. cell death, molecular markers, or behavioral changes).<br>b. For hypothesis-testing studies, specify the primary outcome measure, i.e. the outcome measure that was used to determine the sample size.	a. Methods > animal studies > para. 5th-rest.<br>b. Methods > power analysis.
Statistical methods	7	a. Provide details of the statistical methods used for each analysis, including software used.<br>b. Describe any methods used to assess whether the data met the assumptions of the statistical approach, and what was done if the assumptions were not met.	a. Methods > statistical analysis.<br>b. No methods were used.
Experimental animals	8	a. Provide species-appropriate details of the animals used, including species, strain and substrain, sex, age or developmental stage, and, if relevant, weight.<br>b. Provide further relevant information on the provenance of animals, health/immune status, genetic modification status, genotype, and any previous procedures.	a. Methods > animal studies > 2nd paragraph.<br>b. No genetically modified swine were used.
Experimental procedures	9	For each experimental group, including controls, describe the procedures in enough detail to allow others to replicate them, including:<br>a. What was done, how it was done and what was used.<br>b. When and how often.<br>c. Where (including detail of any acclimatization periods).<br>d. Why (provide rationale for procedures).	a-d. Methods > animal studies.

Results	10	For each experiment conducted, including independent replications, report:<br>a. Summary/descriptive statistics for each experimental group, with a measure of variability where applicable (e.g. mean and SD, or median and range).<br>b. If applicable, the effect size with a confidence interval.	a. The mean of each group and SD were compared. Methods > Statistical analysis.<br>b. The sample size $n = 5$ was enough for the confidence interval.
Abstract	11	Provide an accurate summary of the research objectives, animal species, strain and sex, key methods, principal findings, and study conclusions.	Manuscript > page 1
Background	12	a. Include sufficient scientific background to understand the rationale and context for the study, and explain the experimental approach.<br>b. Explain how the animal species and model used address the scientific objectives and, where appropriate, the relevance to human biology.	a. Manuscript > Introduction>1st - 3rd paragraphs; Methods> animal studies.<br>b. Methods > animal studies > 1st - 4th paragraphs.
Objectives	13	Clearly describe the research question, research objectives and, where appropriate, specific hypotheses being tested.	Introduction > 3rd paragraph and Results. Methods > power analysis.
Ethical statement	14	Provide the name of the ethical review committee or equivalent that has approved the use of animals in this study, and any relevant license or protocol numbers (if applicable). If ethical approval was not sought or granted, provide a justification.	UNMC IACUC; Protocol No. #22-051-08-EP
Housing and husbandry	15	Provide details of housing and husbandry conditions, including any environmental enrichment.	Methods > animal studies > 2nd paragraph
Animal care and monitoring	16	a. Describe any interventions or steps taken in the experimental protocols to reduce pain, suffering and distress.<br>b. Report any expected or unexpected adverse events.<br>c. Describe the humane endpoints established for the study, the signs that were monitored and the frequency of	a. Methods > animal studies > 2nd and 3rd paragraphs.<br>b. No adverse events were observed.

		monitoring. If the study did not have humane endpoints, state this.	c. The study did not have humane endpoints.
Interpretation/ scientific implications	17	a. Interpret the results, taking into account the study objectives and hypotheses, current theory and other relevant studies in the literature.<br>b. Comment on the study limitations including potential sources of bias, limitations of the animal model, and imprecision associated with the results.	a. Introduction > 1st - 3rd paragraphs.<br>b. Manuscript > discussion > last paragraph
Generalizability / translation	18	Comment on whether, and how, the findings of this study are likely to generalize to other species or experimental conditions, including any relevance to human biology (where appropriate).	Manuscript > discussion. Methods > animal studies.
Protocol registration	19	Provide a statement indicating whether a protocol (including the research question, key design features, and analysis plan) was prepared before the study, and if and where this protocol was registered.	Research designed was discussed and planed. The protocol was not registered.
Data access	20	Provide a statement describing if and where study data are available.	Manuscript
Declaration of interests	21	a. Declare any potential conflicts of interest, including financial and non-financial. If none exist, this should be stated.<br>b. List all funding sources (including grant identifier) and the role of the funder(s) in the design, analysis and reporting of the study.	a. No conflict of interest.<br>b. Manuscript > acknowledgments.

Supplementary Table 9. Summary of experimental setups in previous studies utilizing a non-survivable junctional hemorrhage swine model.

**Ref.**	**Animal Model**	**Weight (kg)**	**JH Injury Model**	**Post Injury Free Bleeding Time (s)**	**Manual Compression Time (min)**	**Observation Period (min)**
This work	Yorkshire Swine	~ 49	Femoral Artery	30	3	180
Arnaud et al.[10]	Yorkshire Swine	~ 35	Femoral Artery	45	5	180
Angus et al.[11]	Yorkshire Swine	~ 45	Femoral Artery	45	3	60
Huang et al.[4]	Yorkshire Swine	~ 45	Subclavian Artery and Vein	30	3	60
Lu et al.[5]	Domestic Swine	~ 40	Femoral Artery	-	-	-
Zhao et al.[6]	Yorkshire Swine	~ 100	Subclavian Artery and Vein	30	3	60
Conley et al.[12]	Yorkshire Swine	~ 35	Femoral Artery	45	3	150
Wang et al.[13]	Yorkshire Swine	~ 34	Femoral Artery	45	5	180
Wu et al.[14]	Bama Miniature Swine	~ 29	Femoral Artery	30	3	180
Li et al.[15]	Yorkshire Swine	~ 37	Femoral Artery	45	3	180

Supplementary Table 10. Comparison of NMA of hemostatic time in swine arterial and vein transection model with all other hemostats reported in the literature known for rapid blood coagulation properties (**Figure 6f**).

**No.**	**Hemostat**	**Hemostasis Time (s)**		**Ref.**
		Mean	s.d.	
**1**	No Treatment	1428.00	685.58	This work
**2**	NMA	0.00	0.00	
**3**	QCG®	852.00	364.77	
**4**	Xstat®	795.00	190.21	
**5**	SiOxMed Hemostatic Matrix®	~ 600.00	-	[11]
**6**	GT25/DA8	348.00	84.00	[4]
**7**	PVA Hemostatic Sponge®	1380.00	318.00	
**8**	GCF-3/CT/TH	210.00	24.60	[5]
**9**	Celox®	600.00	-	
**10**	Surgical Gauze®	2025.20	1051.80	[6]
**11**	Cryogel	106.00	107.00	
**12**	CF	195.00	75.60	[13]
**13**	CS	160.20	34.80	
**14**	Standard Gauze®	296.33	28.57	[14]
**15**	MCSD	48.70	11.87	
**16**	BloodStop iX Battle Matrix®	288.00	150.00	[15]

NMA: nano- and microfiber aerogels; QCG®: QuikClot® combat gauze; GT25/DA8: 25 mg Gelatin/8 mg Dopamine; GCF-3/CT/TH: thrombin-incorporated glutaraldehyde/chitosan/silk fibronin short nanofibers with built in cargo-loaded $CaCO_3$ and protonated tranexamic acid; CF: chitosan fibers; CS: chitosan sponge; MCSD: modified chain-based sponge dressings.

Supplementary Table 11. Comparison of NMA of incidence of post-treatment rebleeding in swine arterial and vein transection model with all other hemostats reported in the literature known for rapid blood coagulation properties (**Figure 6h**).

No.	Hemostat	Incidence (%)	Ref.
**1**	NMA	0	This work
**2**	QuikClot® combat gauze (QCG®)	100.00	
**3**	Xstat®	100.00	
**4**	Woundstat®	62.50	10
**5**	Celox®	50.00	
**6**	X-Sponge®	87.50	
**7**	ACS+®	75.00	
**8**	InstaClot®	75.00	
**9**	Alpha Bandage®	100.00	
**10**	Chitoflex®	75.00	
**11**	FP-21®	100.00	
**12**	Hemcon®	100.00	
**13**	BloodStop®	100.00	
**14**	SD	87.50	
**15**	SiOxMed Hemostatic Matrix®	50.00	11
**16**	Surgical Gauze	100.00	6
**17**	Cryogel	60.00	
**18**	CTG®	50.00	12
**19**	HCG®	25.00	
**20**	CF	100.00	13
**21**	CS	100.00	
**22**	BloodStop iX Battle Matrix®	9.09	15

NMA: nano- and microfiber aerogels; QCG®: QuikClot® combat gauze; SD: standard dressings; CF: chitosan fibers; CS: chitosan sponge.

Supplementary Table 12. Hematologic values during experiments with swine subjected to non-survivable junctional hemorrhage following the administration of tested samples. Source data are provided as a Source Data file.

**Hemoglobin (g/dL)**												
**Time**	**Control**			**QCG®**			**XStat®**			**NMA**		
	Mean	s.d.	*n*	Mean	s.d.	*n*	Mean	s.d.	*n*	Mean	s.d.	*n*
Initial	10.68	1.42	5	11.50	1.45	5	10.64	1.34	5	10.26	1.64	5
15 min	5.25	2.53	4	7.73	0.78	5	7.60	0.90	5	8.22	1.26	5
30 min	N/A	N/A	-	N/A	N/A	-	7.90	1.12	5	8.92	1.56	5
60 min	N/A	N/A	-	N/A	N/A	-	7.90	1.25	5	9.26	1.57	5
120 min	N/A	N/A	-	N/A	N/A	-	8.17	1.56	4	9.52	1.49	5
180 min	N/A	N/A	-	N/A	N/A	-	8.25	1.49	4	9.56	1.62	5
**Hematocrit (%)**												
Initial	N/A	N/A	-	N/A	N/A	-	36.22	5.20	5	35.90	5.54	5
15 min	N/A	N/A	-	N/A	N/A	-	26.16	2.98	5	29.04	4.46	5
30 min	N/A	N/A	-	N/A	N/A	-	27.02	3.92	5	31.14	4.80	5
60 min	N/A	N/A	-	N/A	N/A	-	26.92	4.47	5	32.28	5.03	5
120 min	N/A	N/A	-	N/A	N/A	-	28.28	4.95	4	33.30	4.99	5
180 min	N/A	N/A	-	N/A	N/A	-	28.52	4.92	4	33.34	5.19	5
**Platelet (10^3/μL)**												

Initial	337.4	133.62	5	267.80	100.94	5	259.8	50.76	5	320.20	24.55	5
15 min	254.0	116.41	3	232.0	67.01	4	209.8	18.49	5	270.0	46.04	5
30 min	N/A	N/A	-	N/A	N/A	-	226.0	38.10	5	303.0	68.66	5
60 min	N/A	N/A	-	N/A	N/A	-	216.2	39.75	5	315.8	79.09	5
120 min	N/A	N/A	-	N/A	N/A	-	219.5	44.15	4	318.6	95.05	5
180 min	N/A	N/A	-	N/A	N/A	-	212.25	34.56	4	321.8	97.76	5
**Lactate (mmol/L)**												
Initial	3.33	1.95	4	2.11	0.42	4	3.6	1.46	5	4.08	0.79	4
15 min	NA	NA	-	NA	NA	-	5.52	1.40	5	5.43	0.79	4
30 min	N/A	N/A	-	N/A	N/A	-	5.62	3.03	5	4.15	0.66	4
60 min	N/A	N/A	-	N/A	N/A	-	7.12	5.53	5	3.70	0.81	4
120 min	N/A	N/A	-	N/A	N/A	-	5.92	3.21	4	3.73	0.99	4
180 min	N/A	N/A	-	N/A	N/A	-	6.73	4.49	4	3.60	0.96	4

N/A: not applicable due to subject expired or *n* < 3 due to quantity insufficient, missing specimen, or clotted samples; QCG®: QuikClot® combat gauze; NMA: nano- and microfiber aerogels

Supplementary Table 13. Coagulation summaries in swine experiencing non-survivable junctional hemorrhage following administration of tested samples. Source data are provided as a Source Data file.

Prothrombin Time (s)												
Time (min)	**Control**			**QCG®**			**XStat®**			**NMA**		
	Mean	SD	*n*	Mean	SD	*n*	Mean	SD	*n*	Mean	SD	*n*
0	9.78	1.59	4	10.73	0.61	3	10.64	0.40	5	10.76	0.68	5
15	N/A	N/A	-	N/A	N/A	-	11.34	1.35	5	11.04	0.72	5
30	N/A	N/A	-	N/A	N/A	-	11.36	0.50	5	10.40	0.64	5
60	N/A	N/A	-	N/A	N/A	-	11.14	0.51	5	10.56	0.81	5
120	N/A	N/A	-	N/A	N/A	-	11.35	0.30	4	10.50	0.94	5
180	N/A	N/A	-	N/A	N/A	-	11.35	0.85	4	10.14	0.93	5
Activated Partial Thromboplastin Time (s)												
0	17.13	1.87	4	16.60	0.00	3	16.88	0.97	5	17.70	1.81	5
15	16.00	0.00	3	N/A	N/A	-	17.04	1.69	5	19.26	1.65	5
30	N/A	N/A	-	N/A	N/A	-	18.08	2.13	5	18.40	2.19	5
60	N/A	N/A	-	N/A	N/A	-	17.72	2.09	5	17.15	1.92	4
120	N/A	N/A	-	N/A	N/A	-	63.15	91.25	4	18.00	2.31	4
180	N/A	N/A	-	N/A	N/A	-	63.33	91.13	4	18.00	2.31	4
INR												
0	0.83	0.15	4	0.93	0.06	3	0.92	0.04	5	0.94	0.05	5
15	N/A	N/A	-	N/A	N/A	-	0.98	0.11	5	0.96	0.05	5
30	N/A	N/A	-	N/A	N/A	-	0.98	0.04	5	0.94	0.11	5
60	N/A	N/A	-	N/A	N/A	-	0.96	0.05	5	0.92	0.08	5
120	N/A	N/A	-	N/A	N/A	-	0.98	0.05	4	0.90	0.10	5
180	N/A	N/A	-	N/A	N/A	-	0.97	0.09	4	0.92	0.11	5
Fibrinogen (mg/dL)												
0	114.25	8.96	4	105.67	9.02	3	108.80	19.74	5	118.00	13.60	5
15	N/A	N/A	-	N/A	N/A	-	80.00	12.10	5	103.50	17.75	4
30	N/A	N/A	-	N/A	N/A	-	82.50	7.19	4	106.40	16.16	5

60	N/A	N/A	-	N/A	N/A	-	88.00	13.91	5	112.80	22.55	5
120	N/A	N/A	-	N/A	N/A	-	90.75	14.52	4	119.00	22.66	5
180	N/A	N/A	-	N/A	N/A	-	94.00	13.37	4	118.40	21.62	5

N/A: not applicable due to subject expired or *n* < 3 due to quantity insufficient, missing specimen, or clotted samples; QCG®: QuikClot® combat gauze; NMA: nano- and microfiber aerogels

Supplementary Table 14. Treatment outcomes of NMA on arterial blood gas values. Source data are provided as a Source Data file.

HCO_3 (mEq/L)												
**Time (min)**	**Control**			**QCG®**			**XStat®**			**NMA**		
	Mean	SD	*n*	Mean	SD	*n*	Mean	SD	*n*	Mean	SD	*n*
0	31.40	4.76	3	31.65	3.95	4	29.20	2.28	5	28.60	1.14	5
15	N/A	N/A	-	N/A	N/A	-	27.40	3.58	5	27.00	1.41	4
30	N/A	N/A	-	N/A	N/A	-	27.60	4.61	5	28.80	1.30	5
60	N/A	N/A	-	N/A	N/A	-	26.00	8.12	5	29.75	0.96	4
120	N/A	N/A	-	N/A	N/A	-	28.50	4.20	4	29.40	2.70	5
180	N/A	N/A	-	N/A	N/A	-	28.30	5.85	4	31.25	1.71	4
**PaO_2 (mmHg)**												
0	469.0	32.90	3	436.50	60.27	4	401.40	50.49	5	394.0	48.90	5
15	402.0	89.16	3	N/A	N/A	-	391.80	30.29	5	417.00	49.21	4
30	N/A	N/A	-	N/A	N/A	-	384.00	68.20	5	404.60	39.95	5
60	N/A	N/A	-	N/A	N/A	-	375.20	64.00	5	409.50	54.67	4
120	N/A	N/A	-	N/A	N/A	-	377.75	51.22	4	397.60	38.34	5
180	N/A	N/A	-	N/A	N/A	-	370.30	48.19	4	386.50	58.65	4
**$PaCO_2$ (mmHg)**												
0	51.10	15.40	3	58.70	15.80	4	37.40	7.17	5	40.40	5.55	5
15	23.83	7.05	3	N/A	N/A	-	43.00	8.03	5	45.50	9.26	4
30	N/A	N/A	-	N/A	N/A	-	40.40	6.39	5	42.60	8.62	5
60	N/A	N/A	-	N/A	N/A	-	37.00	14.27	5	41.75	7.41	4
120	N/A	N/A	-	N/A	N/A	-	41.75	9.18	4	40.80	9.12	5
180	N/A	N/A	-	N/A	N/A	-	38.25	10.87	4	45.00	8.04	4
**$EtCO_2$ (mmHg)**												
0	44.80	5.36	5	49.00	11.85	5	38.20	3.96	5	42.20	0.45	5
15	11.75	4.99	4	N/A	N/A	-	41.80	8.87	5	38.60	1.52	5

30	N/A	N/A	-	N/A	N/A	-	36.80	5.81	5	38.40	3.36	5
60	N/A	N/A	-	N/A	N/A	-	30.80	18.03	5	37.60	1.67	5
120	N/A	N/A	-	N/A	N/A	-	37.25	2.75	4	38.00	2.00	5
180	N/A	N/A	-	N/A	N/A	-	35.00	3.37	4	39.60	2.30	5

N/A: not applicable due to subject expired or $n < 3$ due to quantity insufficient, missing specimen, or clotted samples; QCG®: QuikClot® combat gauze; NMA: nano- and microfiber aerogels

Supplementary Table 15. Treatment outcomes of NMA on blood pH. Source data are provided as a Source Data file.

**Time (min)**	**Control**			**QCG®**			**XStat®**			**NMA**		
	Mean	SD	*n*	Mean	SD	*n*	Mean	SD	*n*	Mean	SD	*n*
0	7.41	0.07	3	7.35	0.06	4	7.50	0.05	5	7.47	0.06	5
15	7.43	0.02	3	N/A	N/A	-	7.42	0.05	5	7.40	0.08	4
30	N/A	N/A	-	N/A	N/A	-	7.44	0.06	5	7.45	0.09	5
60	N/A	N/A	-	N/A	N/A	-	7.47	0.06	5	7.47	0.08	4
120	N/A	N/A	-	N/A	N/A	-	7.45	0.08	4	7.48	0.09	5
180	N/A	N/A	-	N/A	N/A	-	7.48	0.07	4	7.46	0.08	4

N/A: not applicable due to subject expired or $n < 3$ due to quantity insufficient, missing specimen, or clotted samples; QCG®: QuikClot® combat gauze; NMA: nano- and microfiber aerogels

Supplementary Table 16. Body temperature of swine after treatment. Source data are provided as a Source Data file.

**Time (min)**	**Control**			**QCG®**			**XStat®**			**NMA**		
	Mean	SD	*n*	Mean	SD	*n*	Mean	SD	*n*	Mean	SD	*n*
0	36.68	0.67	5	36.58	0.71	5	36.66	0.68	5	36.76	0.86	5
15	36.40	0.56	3	N/A	N/A	-	36.78	0.59	5	36.76	0.87	5
30	N/A	N/A	-	N/A	N/A	-	36.82	0.75	5	36.82	0.84	5
60	N/A	N/A	-	N/A	N/A	-	37.32	0.56	5	37.02	0.77	5
120	N/A	N/A	-	N/A	N/A	-	37.35	0.31	4	37.36	0.81	5
180	N/A	N/A	-	N/A	N/A	-	37.63	0.49	4	37.56	0.63	5

N/A: not applicable due to subject expired or $n < 3$ due to quantity insufficient, missing specimen, or clotted samples; QCG®: QuikClot® combat gauze; NMA: nano- and microfiber aerogels

Supplementary Figures

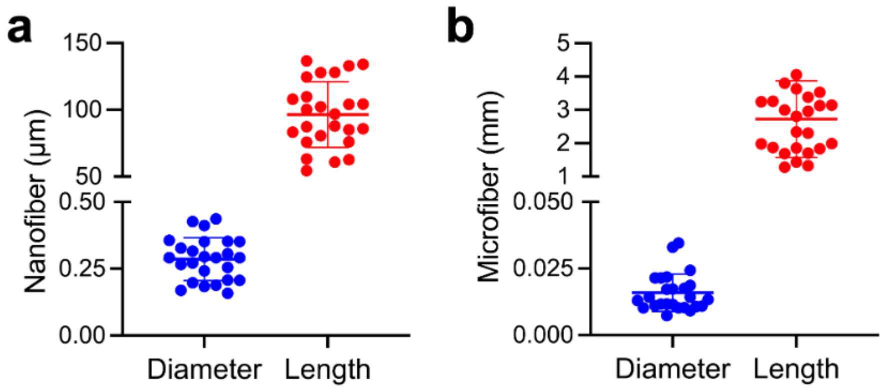

Supplementary Fig. 1| a, Diameter and length of short PLA nanofibers. **b,** Diameter and length of short PCL microfibers. Data were presented as mean ± s.d., n = 25 samples. Source data are provided as a Source Data file.

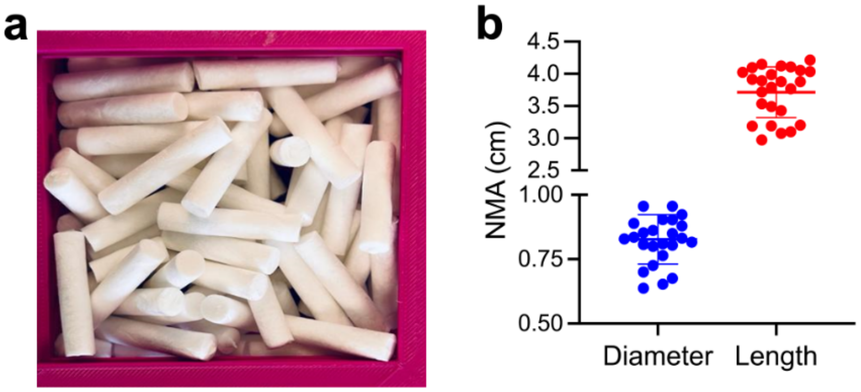

Supplementary Fig. 2| a, Photographs of NMA. **b,** Diameter and length of NMA. Data were presented as mean ± s.d., $n = 25$ samples. NMA: nano- and microfiber aerogels. Source data are provided as a Source Data file.

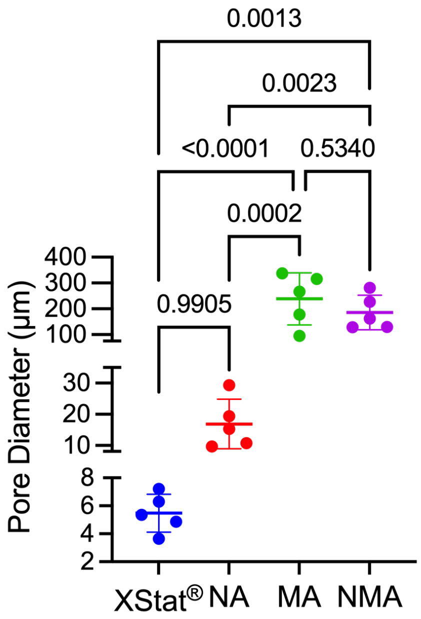

Supplementary Fig. 3| Pore diameter of NA, MA, NMA, and XStat®. Data are presented as mean ± s.d., $n = 5$ samples. The significant difference was detected by one-way ANOVA with Tukey's multiple comparisons test. NA: nanofiber aerogel. MA: microfiber aerogel. NMA: nano- and microfiber aerogels. Source data are provided as a Source Data file.

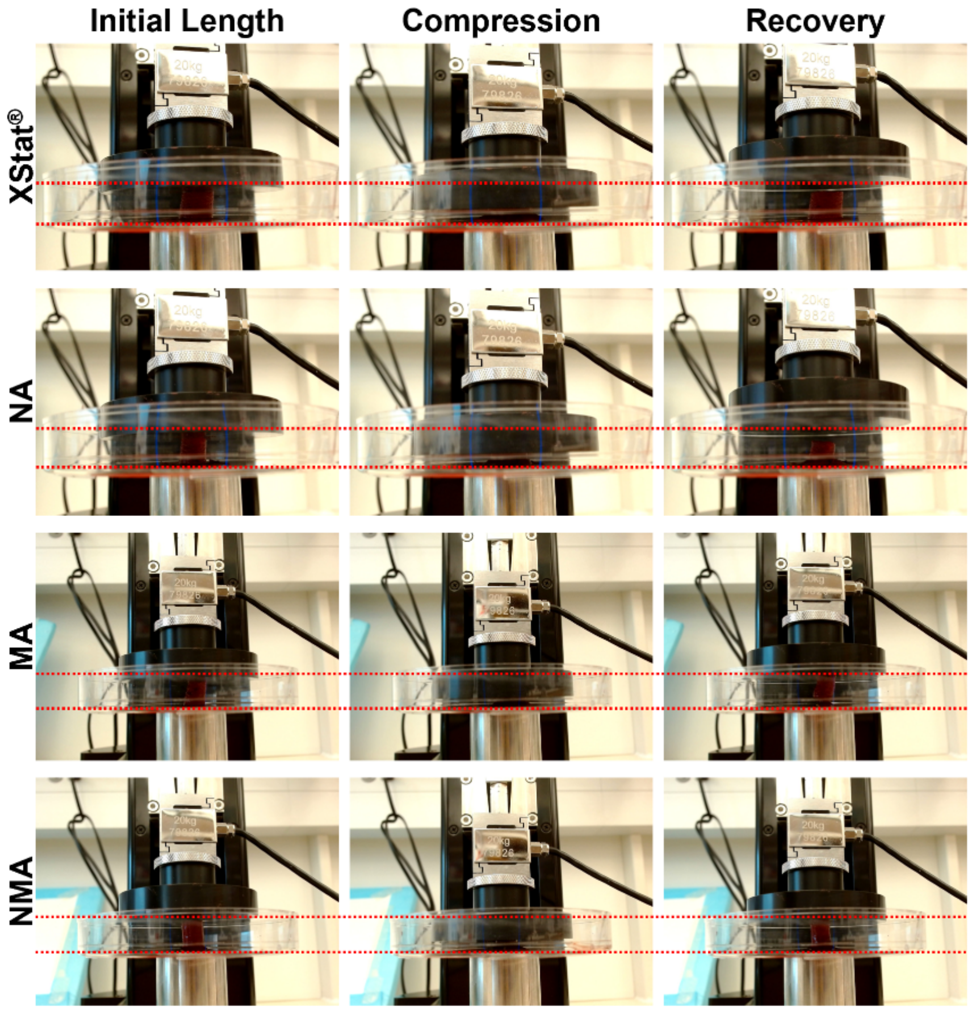

Supplementary Fig. 4| Photographs illustrating the compression and recovery of XStat®, NA, MA, and NMA materials after absorbing human blood at 90% compressive strain. The red dashed lines, with a diameter corresponding to the initial material length, highlight the extent of recovery loss after compression. NA: nanofiber aerogel. MA: microfiber aerogel. NMA: nano- and microfiber aerogels.

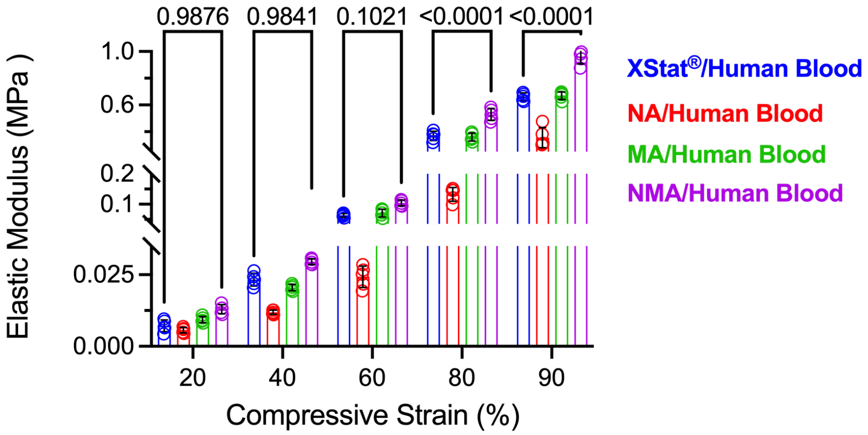

Supplementary Fig. 5| Elastic modulus of different samples under 20%, 40%, 60%, 80%, and 90% compressive strain in human blood. Data are presented as mean ± s.d., n = 5 samples. The significant difference was detected by two-way ANOVA with Tukey's multiple comparisons test. NA: nanofiber aerogels; MA: microfiber aerogels; NMA: nano- and microfiber aerogels. Source data are provided as a Source Data file.

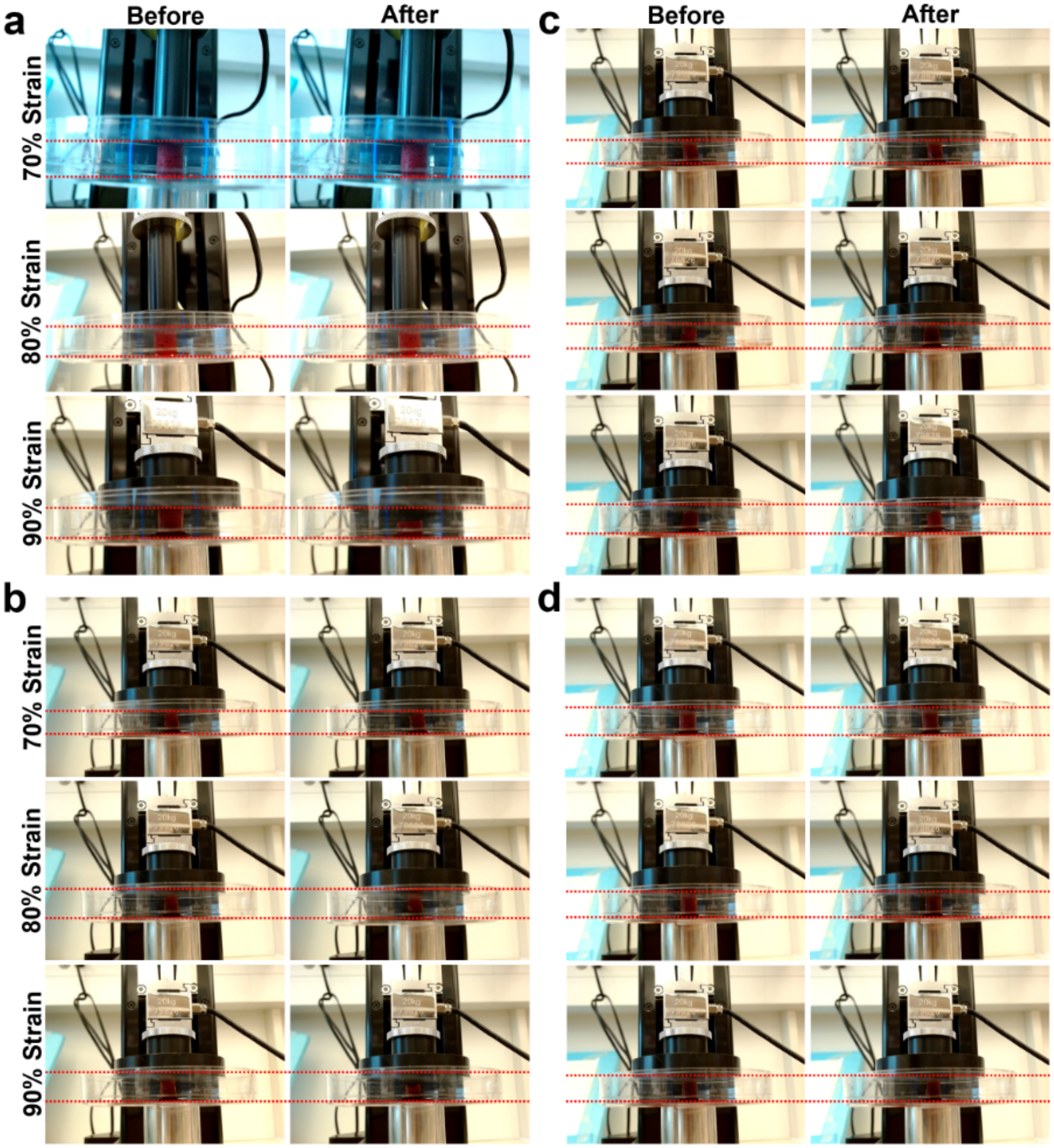

Supplementary Fig. 6| a-d, Photographs showing the length of XStat®(a), NA (b), MA (c), and NMA (d) materials before and after 5 cycles of compression and relaxation at 70%, 80%, and 90% compressive strain following absorption of human blood. The red dashed lines, with a diameter matching the initial material length, indicate the recovery loss observed after cyclic compression. NA: nanofiber aerogel; MA: microfiber aerogel; NMA: nano- and microfiber aerogels.

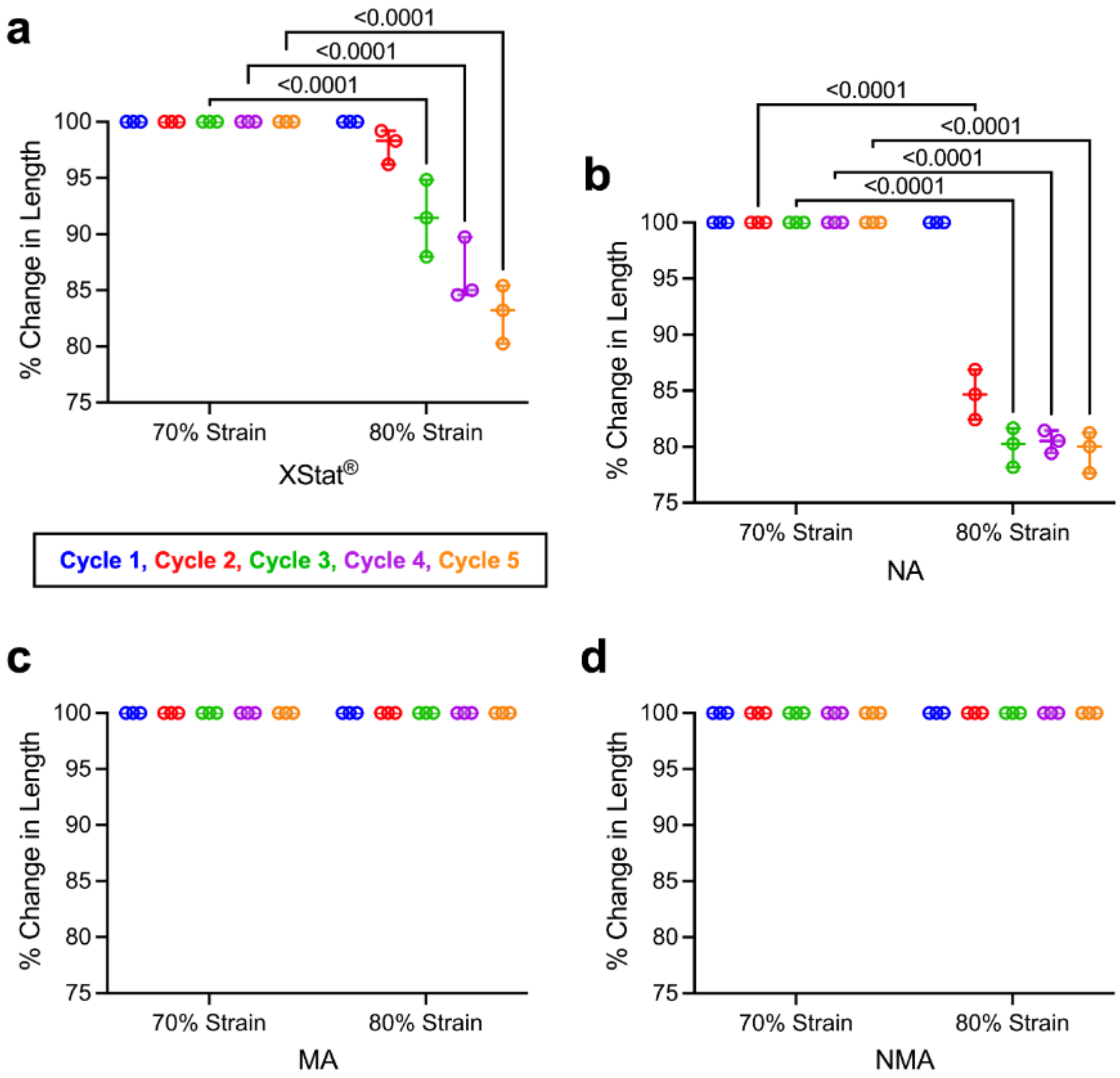

Supplementary Fig. 7| a-d, Changes in length of XStat® (**a**), NA (**b**), MA (**c**), and NMA (**d**) during cyclic compression-relaxation test at 70% and 80% compressive strains, respectively. Data are presented as mean ± s.d., $n = 3$ samples. Two-way ANOVA detected the significant difference with Tukey's multiple comparisons test. NA: nanofiber aerogels; MA: microfiber aerogels; NMA: nano- and microfiber aerogels. Source data are provided as a Source Data file.

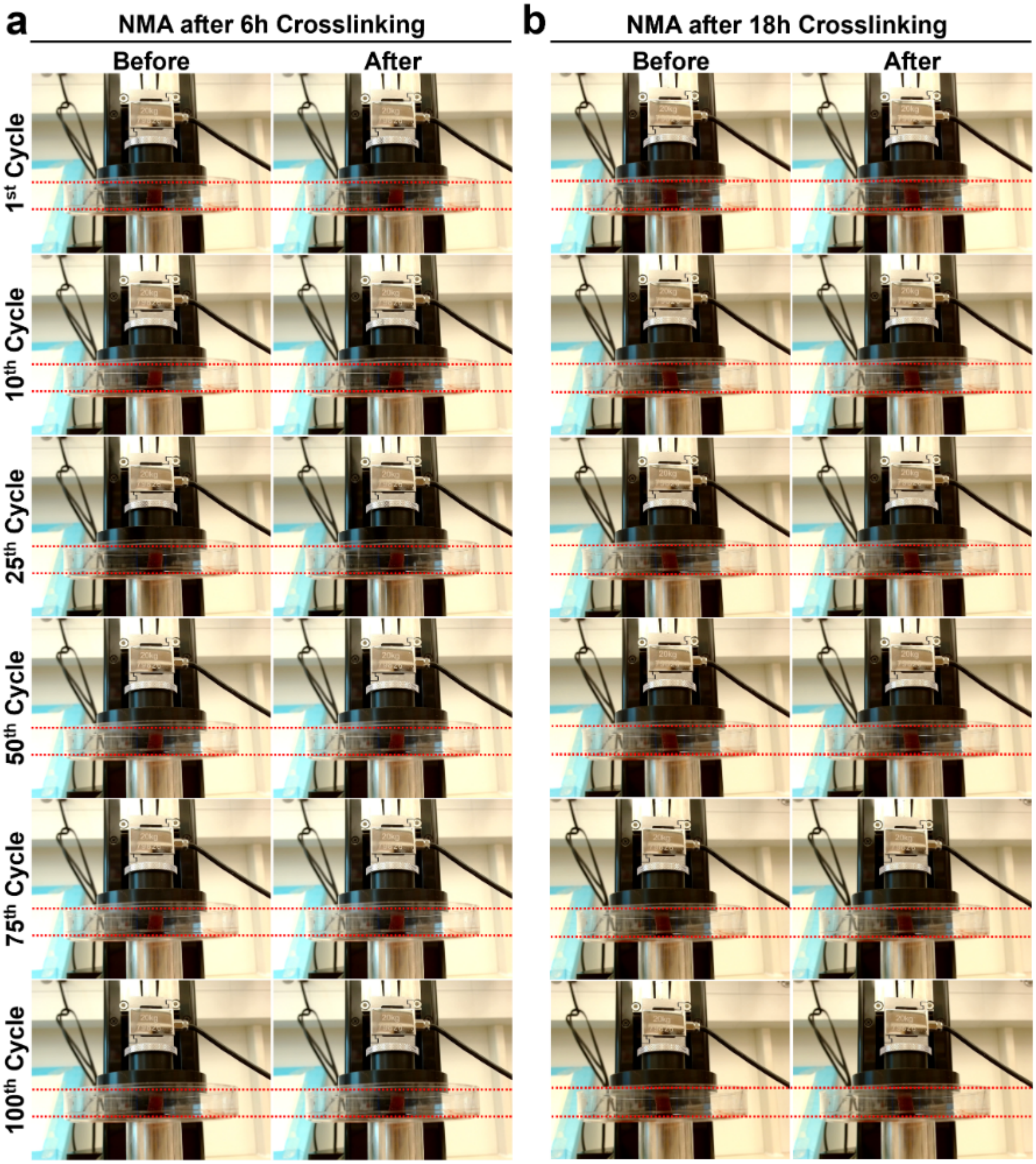

Supplementary Fig. 8| a,b, Photographs showing the length of NMA materials prepared with 6h (a) and 18h (b) crosslinking, before and after the 1st, 10th, 25th, 50th, 75th, and 100th cycles of compression and relaxation at 90% compressive strain following human blood absorption. The red dashed lines, with a diameter corresponding to the initial material length, highlight the recovery loss after cyclic compression. NMA: nano- and microfiber aerogels.

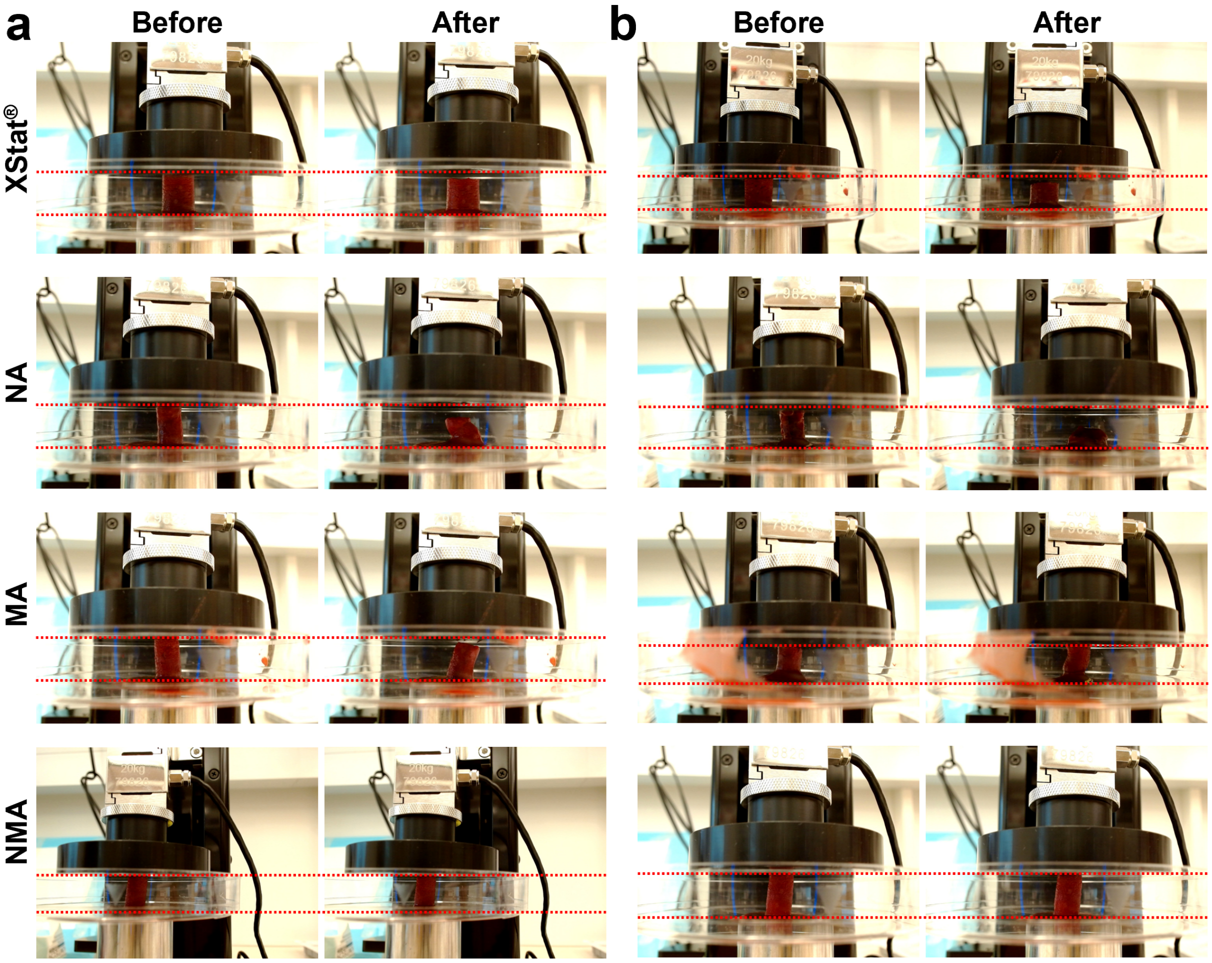

Supplementary Fig. 9| a,b, Photographs showing the length of XStat®, NA, MA, and NMA materials before and after compression and relaxation during testing for toughness (a) as well as elastic energy and resilience (b), following the absorption of human blood. The red dashed lines, with a diameter matching the initial material length, indicate the recovery loss observed after cyclic compression. NA: nanofiber aerogel; MA: microfiber aerogel; NMA: nano- and microfiber aerogels.

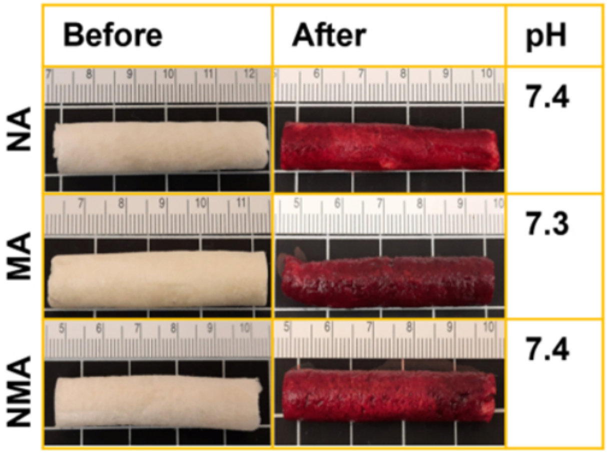

Supplementary Fig. 10| Representative photographs showing aerogels before and after blood absorption. Right column represents the pH values of each group. NA: nanofiber aerogels; MA: microfiber aerogels; NMA: nano- and microfiber aerogels.

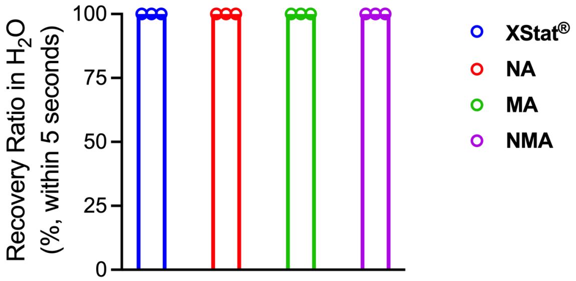

Supplementary Fig. 11| Shape recovery ratios of samples in water within 5 s. Data are presented as mean ± s.d., $n = 3$ samples. NA: nanofiber aerogels; MA: microfiber aerogels; NMA: nano- and microfiber aerogels. Source data are provided as a Source Data file.

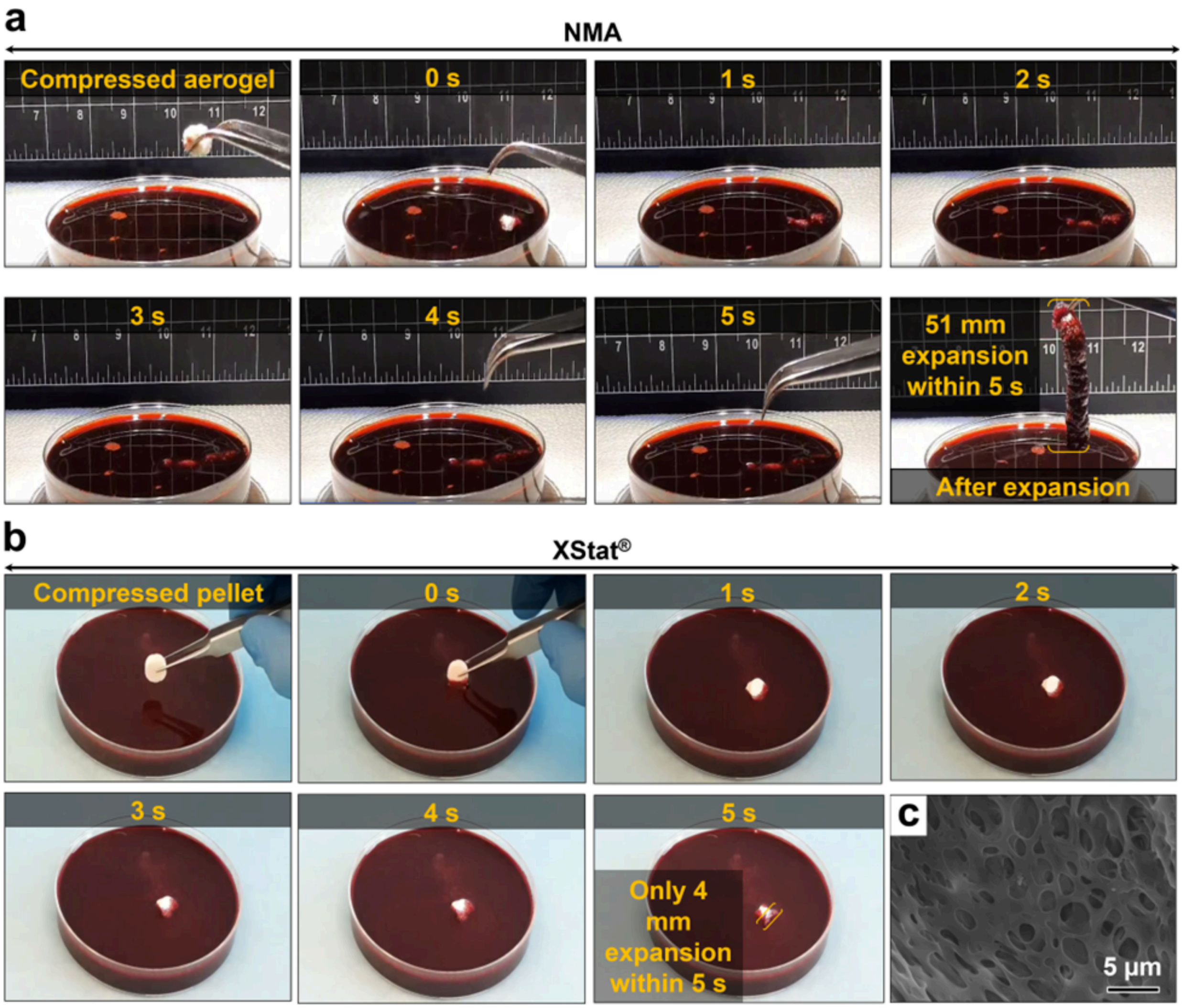

Supplementary Fig. 12| a,b, Photographs showing blood-triggered shape recovery of NMA (**a**) and XStat® (**b**). **c,** SEM image showing the cross section of XStat®. NMA: nano- and microfiber aerogels.

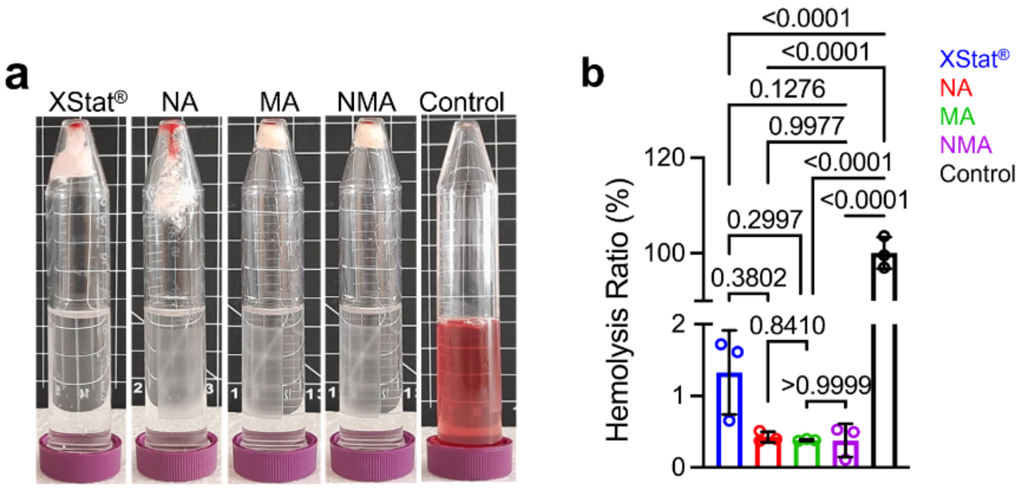

Supplementary Fig. 13| a, Visual representation of the hemolytic activity assay for NA, MA, and NMA, with XStat® as the positive control and water as the negative control. **b,** Hemolysis ratios of the tested samples, presented as mean ± s.d., *n* = 3 samples. Statistical significance was determined by ordinary one-way ANOVA followed by Tukey's multiple comparison tests. NA: nanofiber aerogels; MA: microfiber aerogels; NMA: nano- and microfiber aerogels. Source data are provided as a Source Data file.

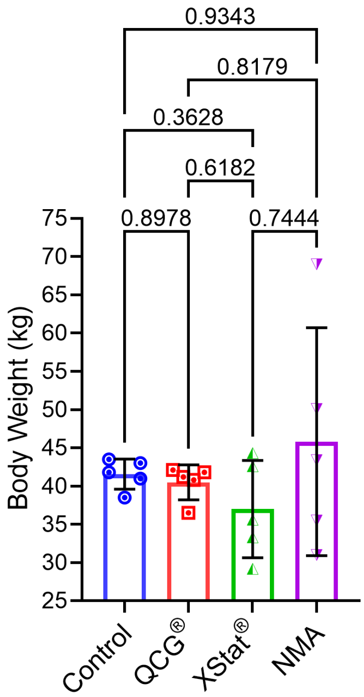

Supplementary Fig. 14| The body weight of Yorkshire Swine used for the animal study. Data were presented as mean ± s.d., n = 5 swine. Statistical significance was determined by ordinary one-way ANOVA followed by Tukey's multiple comparison tests. QCG®: QuikClot® combat gauze; NMA: nano- and microfiber aerogels. Source data are provided as a Source Data file.

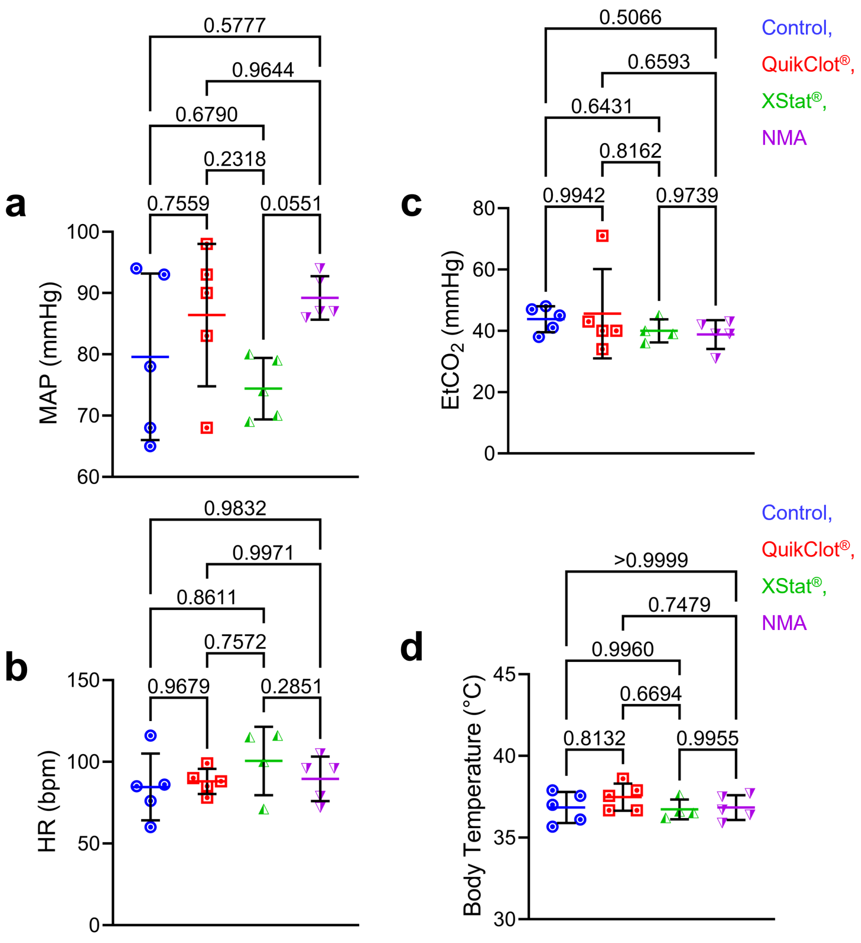

Supplementary Fig. 15| Baseline vitals of Yorkshire swine. **a.** Initial mean arterial pressure (MAP). Data were presented as mean ± s.d., *n* = 5 swine. **b,** Initial heart rate (HR). Data were presented as mean ± s.d., *n* = 4 swine for XStat®, *n* = 5 swine for rest of the groups. **c,** Initial end-tidal carbon dioxide ($EtCO_2$). Data were presented as mean ± s.d., *n* = 4 swine for XStat®, *n* = 5 swine for rest of the groups. **d,** Initial body temperatures. Data were presented as mean ± s.d., *n* = 4 swine for XStat®, *n* = 5 swine for rest of the groups. Statistical significances in **a-d** were determined by ordinary one-way ANOVA followed by Tukey's multiple comparison tests. QCG®: QuikClot® combat gauze; NMA: nano- and microfiber aerogels. Source data are provided as a Source Data file.

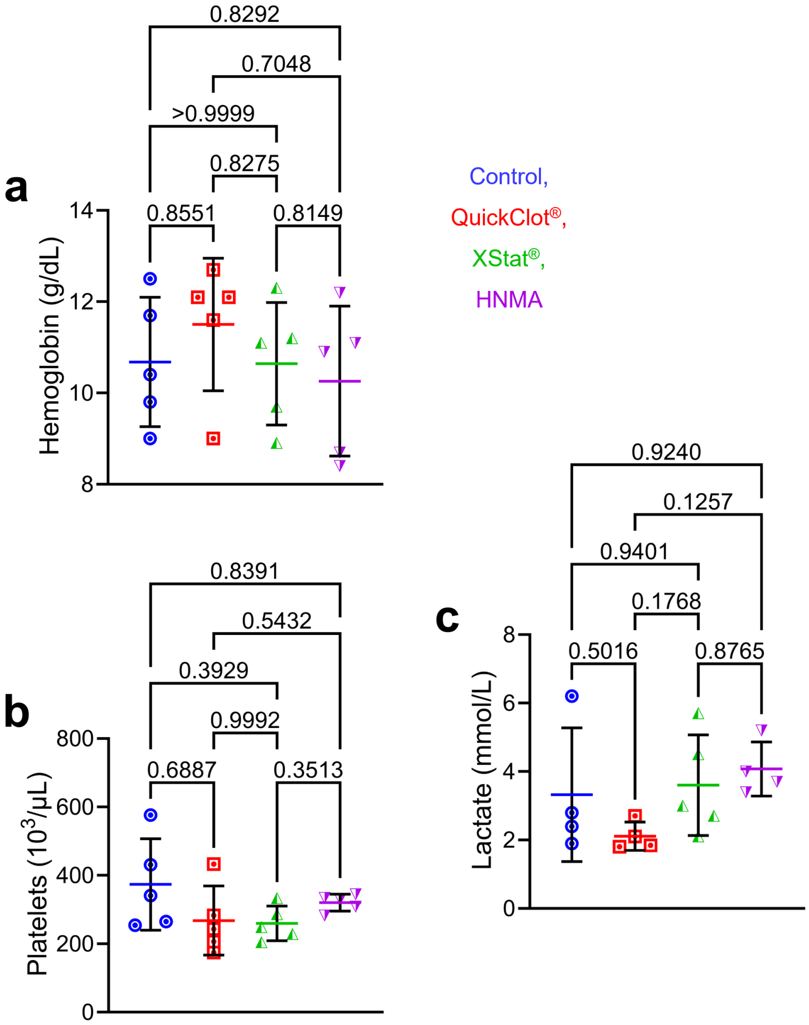

Supplementary Fig. 16| Baseline hematological values of Yorkshire Swine. **a,** Initial hemoglobin levels. Data were presented as mean ± s.d., *n* = 5 swine. **b,** Initial platelets levels. Data were presented as mean ± s.d., *n* = 5 swine. c, Initial lactate levels. Data were presented as mean ± s.d., *n* = 5 swine for XStat® and *n* = 4 swine for the rest of the groups. Statistical significances in **a-c** were determined by ordinary one-way ANOVA followed by Tukey's multiple comparison tests. QCG®: QuikClot® combat gauze; NMA: nano- and microfiber aerogels. Source data are provided as a Source Data file.

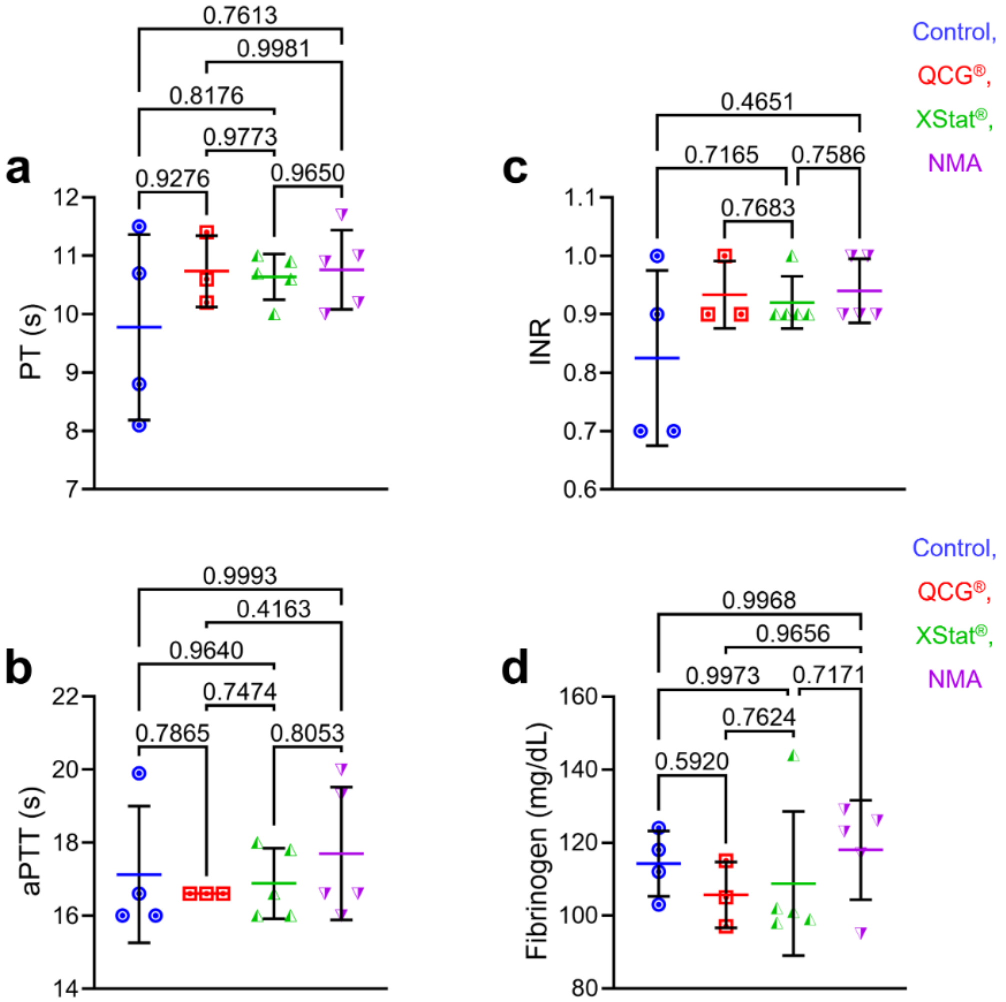

Supplementary Fig. 17| Baseline coagulation data of Yorkshire Swine. **a,** Normal prothrombin time (PT). **b,** Normal activated partial thromboplastin time (aPTT). **c,** Normal international normalized ratio. **d,** Normal fibrinogen levels. Data in **a-d** were presented as mean ± s.d., n = 4 swine for Control, and n = 3 swine for QCG®, and n = 5 swine for XStat® and NMA. Statistical significance in **a-d** were determined by ordinary one-way ANOVA followed by Tukey's multiple comparison tests. QCG®: QuikClot® combat gauze; NMA: nano- and microfiber aerogels. Source data are provided as a Source Data file.

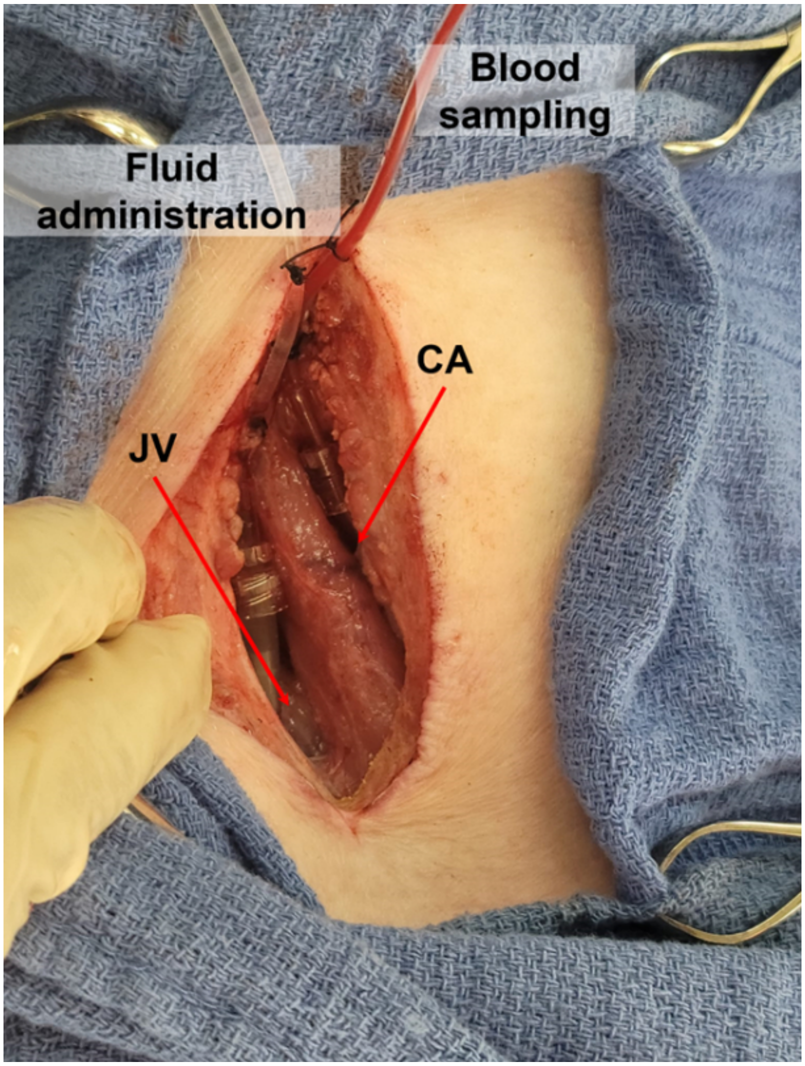

Supplementary Fig. 18| Representative photograph showing surgical placement of a carotid arterial (CA) catheter for pressure monitoring and blood sampling and a jugular venous (JV) catheter for fluid and medication administration via surgical cutdown in the right or left neck.

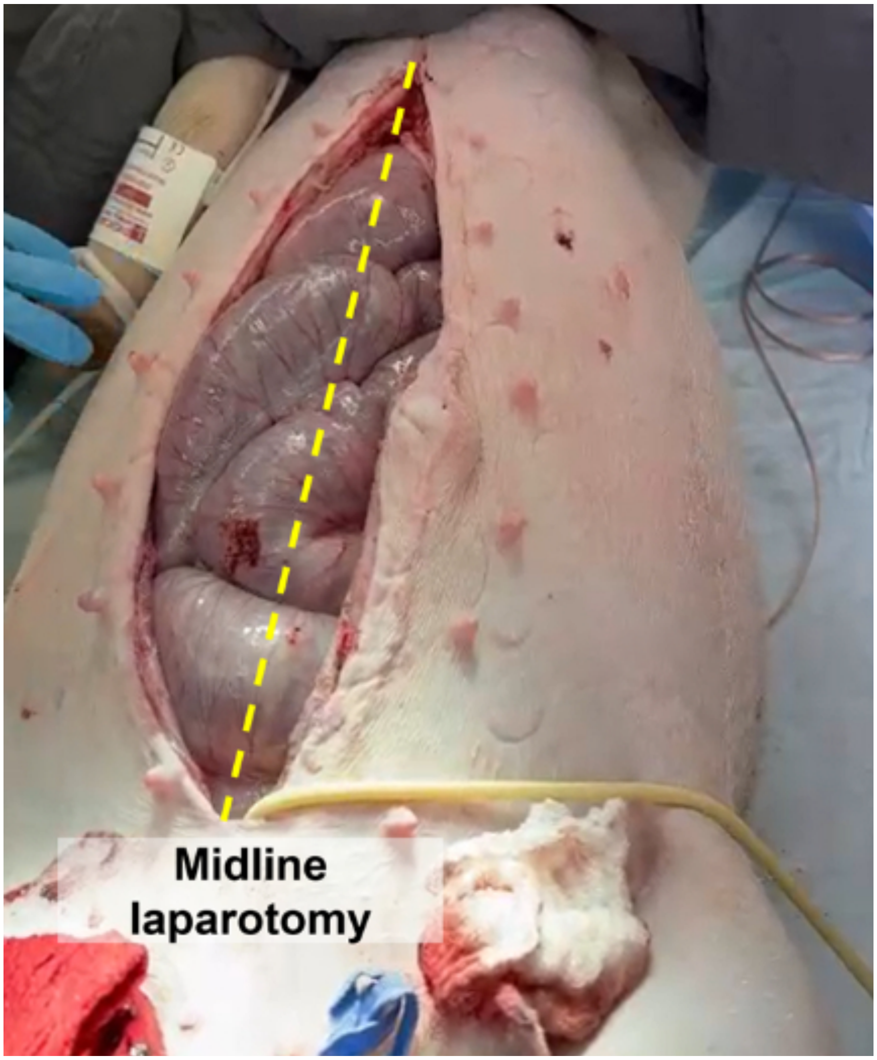

Supplementary Fig. 19| Representative photograph showing the midline laparotomy in Yorkshire Swine.

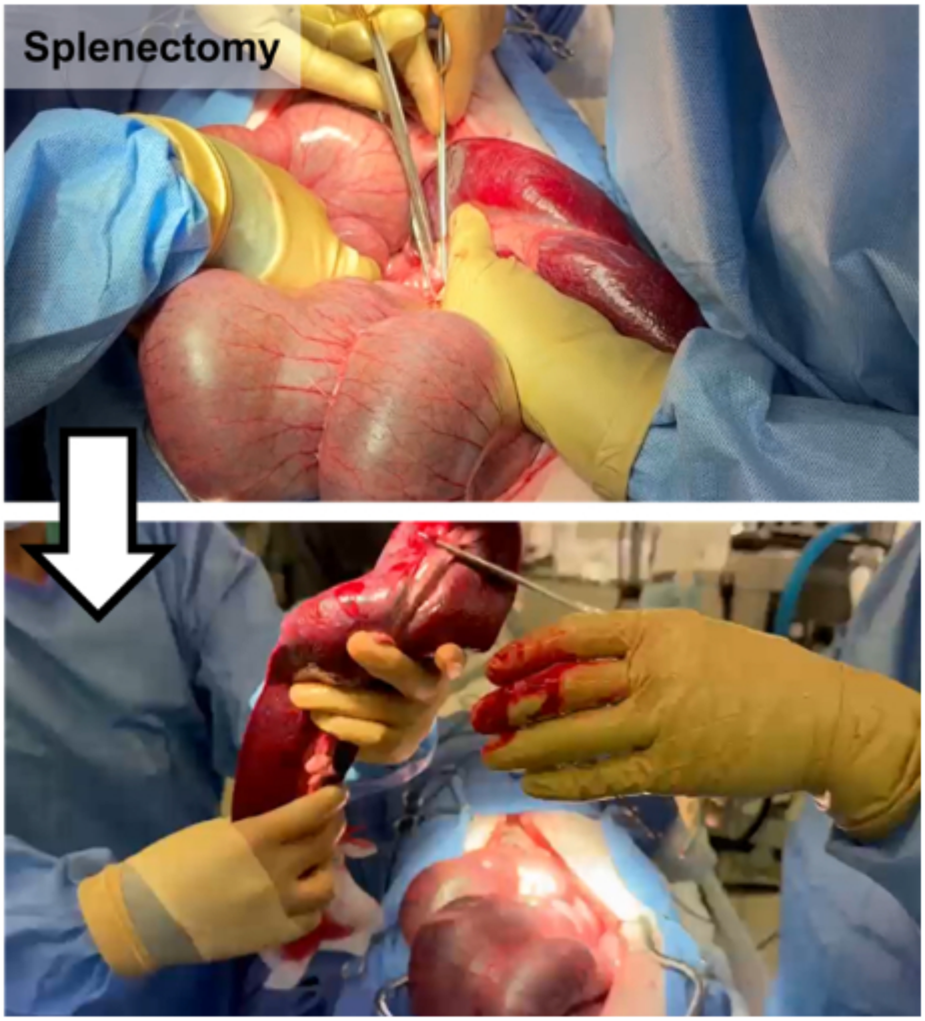

Supplementary Fig. 20| Representative photograph showing the splenectomy performed in Yorkshire Swine to minimize autotransfusion by the contractile porcine spleen.

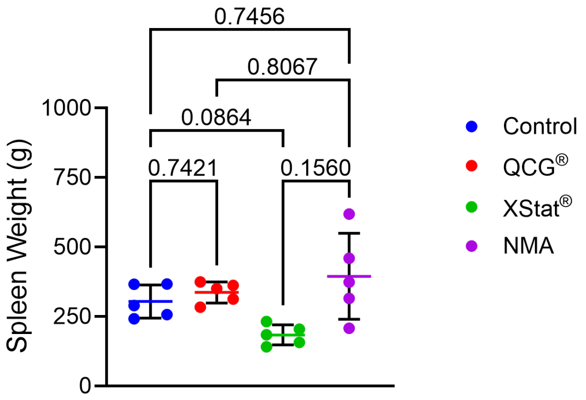

Supplementary Fig. 21| The spleen weight of Yorkshire Swine used for the animal study. Data are presented as mean ± s.d., n = 5 swine. Statistical significance was determined by ordinary one-way ANOVA followed by Tukey's multiple comparison tests. QCG®: QuikClot® combat gauze; NMA: nano- and microfiber aerogels. Source data are provided as a Source Data file.

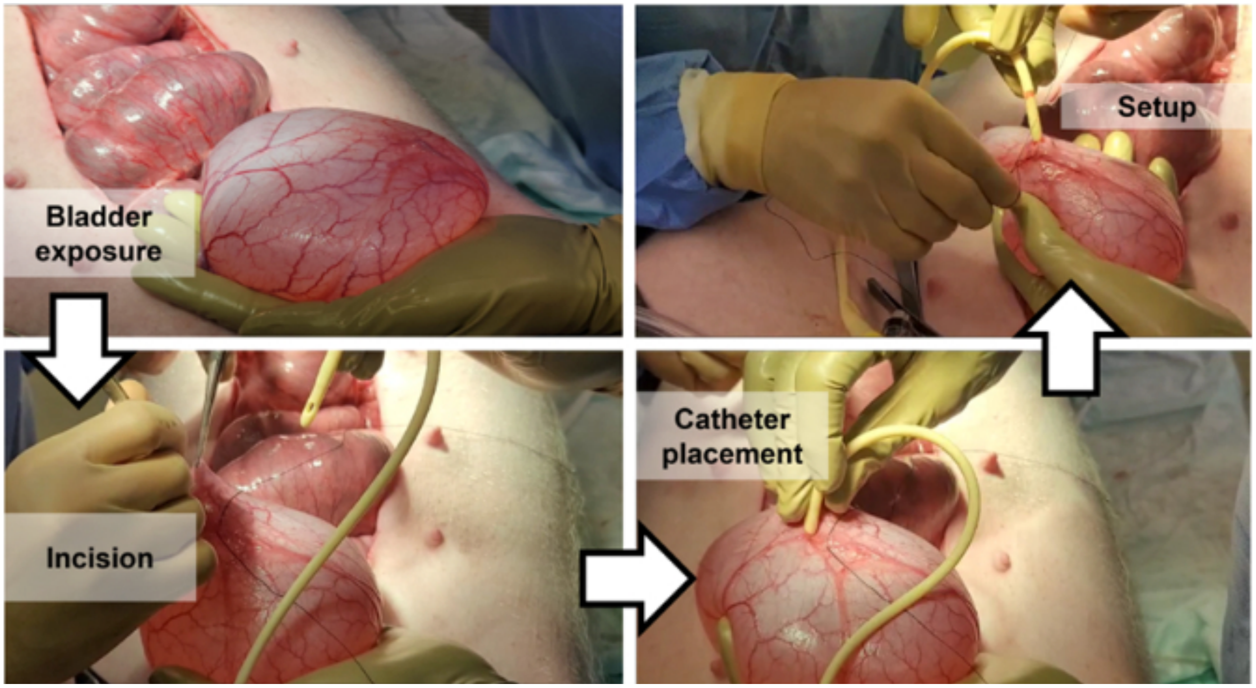

Supplementary Fig. 22| Representative photographs showing bladder decompression procedure through cystostomy in Yorkshire swine to prevent an overly large porcine urinary bladder from exerting pressure on the nearby injury site.

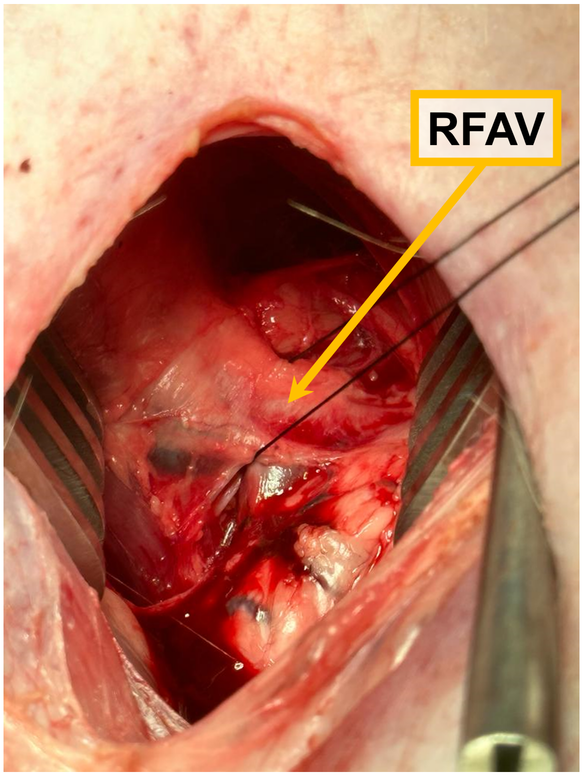

Supplementary Fig. 23| Photographs showing the right femoral arteries of Yorkshire swine. RFAV: right femoral artery and vein.

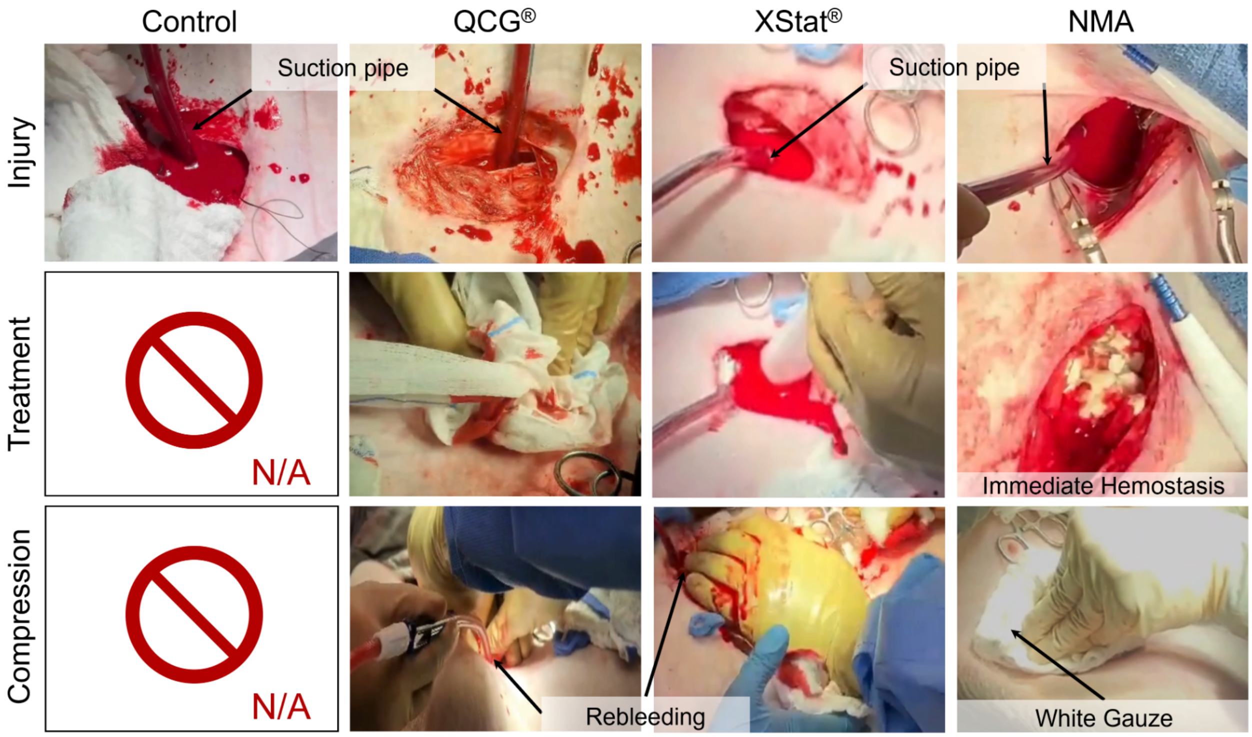

Supplementary Fig. 24| Photographs showing the immediate hemostasis of NMA treatment in a nonsurvivable swine femoral artery/vein hemorrhage model. QCG®: QuikClot® combat gauze; NMA: nano- and microfiber aerogels. N/A: none of the subjects survive.

	Animal ID	Continuous Bleeding	Incidence of Rebleeding		No Bleeding After Treatment
			Single	Multiple	
No Treatment	Swine 1	✓	N/A	N/A	N/A
	Swine 2	✓	N/A	N/A	N/A
	Swine 3	✓	N/A	N/A	N/A
	Swine 4	✓	N/A	N/A	N/A
	Swine 5	✓	N/A	N/A	N/A
QCG®	Swine 6	✗		✓	✗
	Swine 7	✗		✓	✗
	Swine 8	✗		✓	✗
	Swine 9	✗		✓	✗
	Swine 10	✗		✓	✗
XStat®	Swine 11	✗		✓	✗
	Swine 12	✗		✓	✗
	Swine 13	✗		✓	✗
	Swine 14	✗		✓	✗
	Swine 15	✗		✓	✗
NMA	Swine 16	✗	✗	✗	✓
	Swine 17	✗	✗	✗	✓
	Swine 18	✗	✗	✗	✓
	Swine 19	✗	✗	✗	✓
	Swine 20	✗	✗	✗	✓

Supplementary Fig. 25| Summary of bleeding incidence in swine experiencing lethal junctional hemorrhage after different treatments. Control group received no treatment. QCG®: QuikClot® combat gauze; NMA: nano- and microfiber aerogels. N/A represent not applicable.

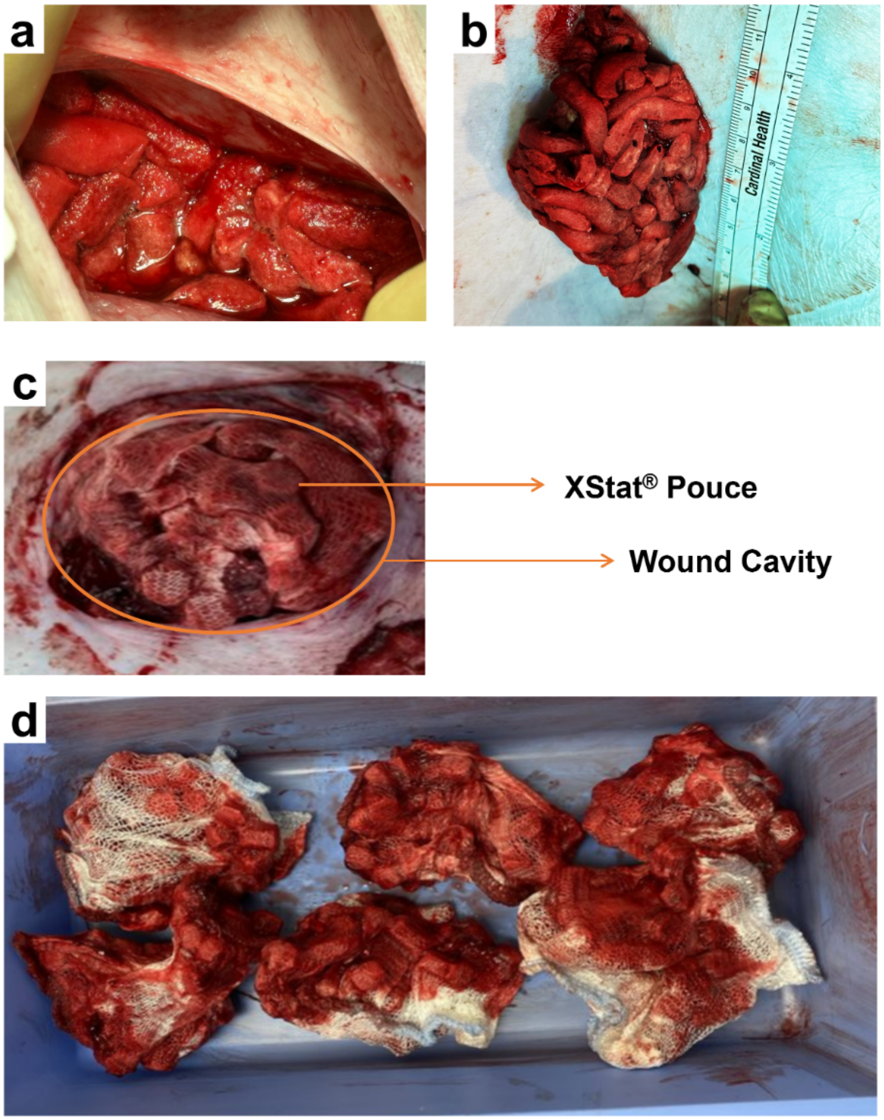

Supplementary Fig. 26| a,b, Images depicting NMA post-treatment before (**a**) and after (**b**) removing the sample from the wound. **c,** XStat® before removing from the wound. **d,** XStat® pouches after removing from the wound. NMA: nano- and microfiber aerogels.

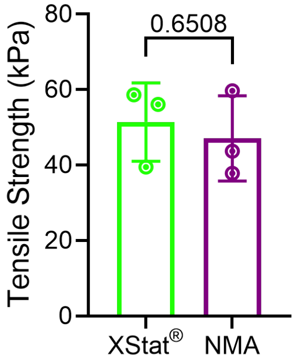

Supplementary Fig. 27| Tensile strength of NMA and XStat® materials following blood absorption. Data were analyzed using an unpaired Student's t-test for statistical significance (ns = $p > 0.05$), with results presented as mean ± standard deviation (n = 3 samples). Source data are provided as a Source Data file.

References

1. Chen, S., Carlson, M. A., Zhang, Y. S., Hu, Y. & Xie, J. Fabrication of injectable and superelastic nanofiber rectangle matrices (“peanuts”) and their potential applications in hemostasis. *Biomaterials* **179,** 46–59 (2018).
2. Du, X. *et al.* Microchannelled alkylated chitosan sponge to treat noncompressible hemorrhages and facilitate wound healing. *Nat. Commun.* **12,** 4733 (2021).
3. Li, M., Zhang, Z., Liang, Y., He, J. & Guo, B. Multifunctional tissue-adhesive cryogel wound dressing for rapid nonpressing surface hemorrhage and wound repair. *ACS Appl. Mater. Interfaces* **12,** 35856–35872 (2020).
4. Huang, Y. *et al.* Degradable gelatin-based IPN cryogel hemostat for rapidly stopping deep noncompressible hemorrhage and simultaneously improving wound healing. *Chem. Mater.* **32,** 6595–6610 (2020).
5. Lu, B. *et al.* Bioinspired hemostatic strategy via pulse ejections for severe bleeding wounds. *Research* **6,** 0150 (2023).
6. Zhao, X. *et al.* Injectable dry cryogels with excellent blood-sucking expansion and blood clotting to cease hemorrhage for lethal deep-wounds, coagulopathy and tissue regeneration. *Chem. Eng. J.* **403,** 126329 (2021).
7. Zhao, X., Guo, B., Wu, H., Liang, Y. & Ma, P. X. Injectable antibacterial conductive nanocomposite cryogels with rapid shape recovery for noncompressible hemorrhage and wound healing. *Nat. Commun.* **9,** 2784 (2018).
8. Yang, G. *et al.* Super-elastic carbonized mushroom aerogel for management of uncontrolled hemorrhage. *Adv. Sci.* **10,** 2207347 (2023).
9. Jiang, T. *et al.* Superporous sponge prepared by secondary network compaction with enhanced permeability and mechanical properties for non-compressible hemostasis in pigs. *Nat. Commun.* **15,** 5460 (2024).
10. Arnaud, F., Teranishi, K., Tomori, T., Carr, W. & McCarron, R. Comparison of 10 hemostatic dressings in a groin puncture model in swine. *J. Vasc. Surg.* **50,** 632-639.e1 (2009).
11. Angus, A. A. *et al.* Testing and evaluation of a novel hemostatic matrix in a swine junctional hemorrhage model. *J. Surg. Res.* **291,** 452–458 (2023).

12. Conley, S. P., Littlejohn, L. F., Henao, J., DeVito, S. S. & Zarow, G. J. Control of junctional hemorrhage in a consensus swine model with hemostatic gauze products following minimal training. *Mil. Med.* **180,** 1189–1195 (2015).
13. Wang, Y.-H. *et al.* Evaluation of chitosan-based dressings in a swine model of artery-injury-related shock. *Sci. Rep.* **9,** 14608 (2019).
14. Wu, W. *et al.* A modified chain-based sponge dressing controls junctional hemorrhage in the tactical combat casualty care simulation of pigs. *Scand. J. Trauma Resusc. Emerg. Med.* **31,** 75 (2023).
15. Li, H. *et al.* Comparison of topical hemostatic agents in a swine model of extremity arterial hemorrhage: BloodSTOP iX Battle Matrix vs. QuikClot Combat Gauze. *Int. J. Mol. Sci.* **17,** 545 (2016).
