# Supplementary material for: Bicomponent nano- and microfiber aerogels for effective management of junctional hemorrhage
Source: Nat Commun. 2025 Mar 11;16:2403. doi: 10.1038/s41467-025-57836-0 (PMC11893793; doi:10.1038/s41467-025-57836-0)
Supplement: Supplementary file 2 — Description of Additional Supplementary Files [file 41467_2025_57836_MOESM2_ESM.pdf]

Description of Additional Supplementary Files

File Name: Supplementary Movie 1

Description: Video showing Micro-CT dynamic scanning of NA.

File Name: Supplementary Movie 2

Description: Video showing Micro-CT dynamic scanning of MA.

File Name: Supplementary Movie 3

Description: Video showing Micro-CT dynamic scanning of NMA.

File Name: Supplementary Movie 4

Description: Video showing dry weight differences between XStat® and NMA.

File Name: Supplementary Movie 5

Description: Video showing simulation of stress distribution in NMA.

File Name: Supplementary Movie 6

Description: Video showing shape recovery of XStat® in water.

File Name: Supplementary Movie 7

Description: Video showing shape recovery of NMA in water.

File Name: Supplementary Movie 8

Description: Video showing shape recovery of XStat® in human blood.

File Name: Supplementary Movie 9

Description: Video showing shape recovery of NMA in human blood.

File Name: Supplementary Movie 10

Description: Video showing wettability of NMA in water and human blood.

File Name: Supplementary Movie 11

Description: Video showing reversible fluid absorption properties of NMA.

File Name: Supplementary Movie 12

Description: Video showing splenectomy in a nonsurvivable swine junctional hemorrhage model.

File Name: Supplementary Movie 13

Description: Video showing cystostomy in a nonsurvivable swine junctional hemorrhage model.

File Name: Supplementary Movie 14

Description: Video showing midline laparotomy incision closure and blanket coverage to prevent hypothermia.

File Name: Supplementary Movie 15

Description: Video showing femoral artery and vein exposure and identification in a Yorkshire swine.

File Name: Supplementary Movie 16

Description: Video showing euthanasia process in a swine junctional hemorrhage model.

File Name: Supplementary Movie 17

Description: Video showing hemostasis of the untreated (control) group in a nonsurvivable swine junctional hemorrhage model.

File Name: Supplementary Movie 18

Description: Video showing hemostasis of the QuikClot® Combat Gauze group in a nonsurvivable swine junctional hemorrhage model.

File Name: Supplementary Movie 19

Description: Video showing hemostasis of the XStat® group in a nonsurvivable swine junctional hemorrhage model.

File Name: Supplementary Movie 20

Description: Video showing hemostasis of the NMA group in a nonsurvivable swine junctional hemorrhage model.

File Name: Supplementary Movie 21

Description: Video showing incidence of rebleeding during compression after treatment with QuikClot® Combat Gauze in a nonsurvivable swine junctional hemorrhage model.

File Name: Supplementary Movie 22

Description: Video showing incidence of rebleeding during compression after treatment with XStat® in a nonsurvivable swine junctional hemorrhage model.

File Name: Supplementary Movie 23

Description: Video showing no incidence of rebleeding during compression after treatment with NMA in a nonsurvivable swine junctional hemorrhage model.

File Name: Supplementary Movie 24

Description: Video showing incidence of rebleeding after treatment with QuickClot® Combat Gauze and 3 minutes of manual compression in a nonsurvivable swine junctional hemorrhage model.

File Name: Supplementary Movie 25

Description: Video showing incidence of rebleeding after treatment with XStat® and 3 minutes of manual compression in a nonsurvivable swine junctional hemorrhage model.

File Name: Supplementary Movie 26

Description: Video showing no incidence of rebleeding after treatment with NMA and 3 minutes of manual compression in a nonsurvivable swine junctional hemorrhage model.
